# Epigenomic profiling of glucocorticoid responses identifies *cis*-regulatory disruptions impacting steroid resistance in childhood acute lymphoblastic leukemia

**DOI:** 10.1038/s41375-022-01685-z

**Published:** 2022-08-26

**Authors:** Brennan P. Bergeron, Jonathan D. Diedrich, Yang Zhang, Kelly R. Barnett, Qian Dong, Daniel C. Ferguson, Robert J. Autry, Wenjian Yang, Baranda S. Hansen, Colton Smith, Kristine R. Crews, Yiping Fan, Ching-Hon Pui, Shondra M. Pruett-Miller, Mary V. Relling, Jun J. Yang, Chunliang Li, William E. Evans, Daniel Savic

**Affiliations:** 1 Hematological Malignancies Program and Center for Precision Medicine in Leukemia, St. Jude Children’s Research Hospital, Memphis, TN, USA.; 2 Department of Pharmacy and Pharmaceutical Sciences, St. Jude Children’s Research Hospital, Memphis, TN, USA.; 3 Graduate School of Biomedical Sciences, St. Jude Children’s Research Hospital, Memphis, TN, USA.; 4 Department of Tumor Cell Biology, St. Jude Children’s Research Hospital, Memphis, TN, USA.; 5 Integrated Biomedical Sciences Program, University of Tennessee Health Science Center, Memphis, TN, USA.; 6 Department of Cell and Molecular Biology and Center for Advanced Genome Engineering, St. Jude Children’s Research Hospital, Memphis, TN, USA.; 7 Department of Computational Biology, St. Jude Children’s Research Hospital, Memphis, TN, USA.; 8 Department of Oncology, St. Jude Children’s Research Hospital, Memphis, TN, USA.; 9 These authors contributed equally: Brennan P. Bergeron, Jonathan D. Diedrich.

## Abstract

Glucocorticoids (GCs) are a mainstay of contemporary, multidrug chemotherapy in the treatment of childhood acute lymphoblastic leukemia (ALL), and resistance to GCs remains a major clinical concern. Resistance to GCs is predictive of ALL relapse and poor clinical outcome, and therefore represents a major hurdle limiting further improvements in survival rates. While advances have been made in identifying genes implicated in GC resistance, there remains an insufficient understanding of the impact of *cis*-regulatory disruptions in resistance. To address this, we mapped the gene regulatory response to GCs in two ALL cell lines using functional genomics and high-throughput reporter assays and identified thousands of GC-responsive changes to chromatin state, including the formation of over 250 GC-responsive super-enhancers and a depletion of AP-1 bound *cis*-regulatory elements implicated in cell proliferation and anti-apoptotic processes. By integrating our GC response maps with genetic and epigenetic datasets in primary ALL cells from patients, we further uncovered *cis*-regulatory disruptions at GC-responsive genes that impact GC resistance in childhood ALL. Overall, these data indicate that GCs initiate pervasive effects on the leukemia epigenome, and that alterations to the GC gene regulatory network contribute to GC resistance.

## INTRODUCTION

Acute lymphoblastic leukemia (ALL) is the most common cancer in children, and represents 25% of all childhood cancers [1]. Despite treatment advances, relapsed ALL remains the fifth most common pediatric cancer [1]. Unlike newly diagnosed ALL, relapsed ALL has a low overall survival of only 40% [2], and is commonly characterized by chemotherapeutic drug resistance [3]. Consequently, understanding mechanisms of antileukemic drug resistance is critical for improving overall survival in childhood ALL.

Glucocorticoids (GCs) are a mainstay of contemporary multidrug chemotherapy in ALL and resistance to GCs is predictive of ALL relapse and poor clinical outcome [4–11]. GCs are unique chemotherapeutic agents because they function through activation of the glucocorticoid receptor (GR) transcription factor (TF), which is encoded by the *NR3C1* gene [12]. Once activated by GCs, GR translocates into the nucleus where it binds to glucocorticoid response element (GRE) sequences or forms protein–protein interactions at *cis*-regulatory elements to drive a transcriptional program that leads to leukemic cell apoptosis [12, 13]. Although the exact molecular mechanism is not fully understood, reduced glucose metabolism [14] and metabolic reprogramming [15], or the suppression of B-cell development genes [16] has been suggested.

Previous studies have identified transcriptional signatures and genes implicated in GC resistance in ALL [10,16–19]. Up-regulation of the NALP3 inflammasome was associated with CASP1-mediated cleavage of GR, leading to GC resistance [17], and loss of GR expression through mutation was also uncovered as a mechanism of GC resistance [20]. Another mechanism involves the *BTG1* gene, which is commonly deleted in pediatric ALL and impacts GR autoinduction [21]. Transcriptional co-factors are also implicated in GC resistance. *CREBBP* and *NCOR1* are genes frequently mutated at relapse in ALL, and *CREBBP* mutations impair the activation of GC-responsive genes [22]. *TBL1XR1* gene repression is also observed at relapse and impacts GR chromatin recruitment [23], and decreased expression of SWI/SNF subunits was associated with GC-resistance [24]. Phosphorylation and inactivation of the EHMT1/2 coregulator complex via up-regulation of AURKB at relapse were also shown to negatively impact GC-induced activation of genes that drive cell apoptosis [19].

Because the mechanism of action for GCs involves activation of the GR TF and the impact of transcriptional co-factors on GC resistance, disruption of GR binding sites would also be predicted to impact GC resistance. In support of this, a recent study mapped lymphocyte-specific chromatin accessibility in ALL cells and identified chromatin alterations that correlate with GC resistance, including a GR site within the *BIM* gene locus that harbored enhanced DNA methylation in GC-resistant cells [25]. Apart from this study, there has otherwise been limited investigation on how alterations to GR binding impact GC sensitivity in ALL.

To better understand the gene regulatory responses to GCs in ALL, and the impact of *cis*-regulatory disruptions on GC resistance, we mapped epigenomic responses to GCs in two ALL cell lines (697 and Nalm6) and integrated these maps with genomic data in primary ALL cells from patients. These two ALL cell lines were chosen for technical and biological reasons. They grow well in culture and cellular transfections are more efficient in these cell lines compared to other established ALL cell lines. They are also widely used in ALL research studies and are representative of ALL molecular subtypes that are common in children (697 = TCF3-PBX1 and Nalm6 = DUX4/ERG). Notably, both cell lines are sensitive to GCs, thereby allowing the mapping of gene regulatory effects leading to cellular apoptosis. Because of epigenetic and gene regulatory heterogeneity among diverse ALL subtypes [26], the use of two cell lines that are representative of distinct subtypes additionally provides for a more comprehensive evaluation of the ALL cell GC response. Collectively, our study delineated extensive GC-mediated effects on the chromatin landscape and identified genetic and epigenetic *cis*-regulatory disruptions of GC-responsive genes as a mechanism impacting GC resistance in childhood ALL.

## MATERIALS/SUBJECTS AND METHODS

### Patient samples

Written informed consent was obtained from all patients or their legal guardians. Gene expression, DNA methylation, SNV genotyping and ex vivo drug sensitivity data [17, 18] were collected as part of St. Jude Total Therapy XVI (NCT00549848) [27]. The use of these samples was approved by the institutional review board at St. Jude Children’s Research Hospital. Leukemia blasts were isolated from bone marrow obtained prior to treatment and subject to Ficoll gradient centrifugation. Samples underwent further enrichment by magnetic-activated cell sorting if blast percent was <85%.

### Functional genomics assays

Fast-ATAC was performed [28] on 10,000 fresh cells from ALL cell lines. For RNA-seq, total RNA was purified from patient samples using the Norgen Total RNA Purification Kit (Norgen, 35300). ChIP-seq using anti-GR-alpha (BD, 611227) and anti-H3K27ac (Active Motif, 39133) antibodies was performed as previously described [29, 30]. Fast-ATAC data from primary ALL cells were downloaded from NCBI Gene Expression Omnibus (GSE161501). Additional details are provided in Supplementary Methods.

### ATAC-STARR-seq

Fast-ATAC transposed DNA from 697 and Nalm6 cells was cloned into the hSTARR-seq_ORI vector (Addgene plasmid #99296). Cells were transfected and treated with vehicle control or prednisolone. Additional details are provided in Supplementary Methods.

### Western blot

Cells were plated 1 × 10^6^ cells/mL and treated with prednisolone (697 = 10 μM; Nalm6 = 5 μM) for 24 h or vehicle control. Cells were collected, washed with PBS and lysed in RIPA Buffer with protease and phosphatase inhibitors. Protein (40μg) was loaded and run on 4–12% Bis-Tris gradient gels, transferred onto PVDF membrane, blocked with 5% Milk in TBST and incubated with primary antibodies overnight at 4°C. The following antibodies were used: TLE1 (Abcam, ab183742), ROR1 (Cell Signaling Technologies, 4102S), Actin (Sigma, A5441). Membranes were washed with TBST and incubated with HRP-conjugated secondary antibody.

### CRISPR/Cas9 genome editing

Gene and HGR knockouts were generated using CRISPR/Cas9 genome editing after transient transfection with precomplexed ribonuclear proteins. Additional details are provided in Supplementary Methods.

### CRISPR interference screens

All sgRNAs and control non-targeting sgRNAs were designed and synthesized by Custom Array, amplified, purified (Qiagen PCR Purification Kit, #28104), cloned into the lentiviral pXPR_003-puro-IRES-CFP vector using Gibson Assembly Cloning Kit (NEB, #E5510S) and packaged into viral particles. Additional details are provided in Supplementary Methods.

### Drug viability assays

Drug viability assays were performed in 96-well plates and treated for 72-h (ALL cell lines) or 96-h (primary ALL cells) with prednisolone as previously outlined [17, 18]. Additional details are provided in Supplementary Methods.

### Luciferase reporter assays

A 300-bp of sequence centered on reference or the alternative allele of rs7045812 was cloned upstream of the minimal promoter in pGL4.23 (Promega, E841A). Nalm6 cells were transfected with constructs using the Neon transfection system (Thermo Fisher, MPK5000). Following an overnight incubation after transfection, cells were treated with 5uM prednisolone or vehicle control for 6 h before luciferase was measured using the Dual Luciferase Reporter Assay System (Promega, E1960).

### Data analysis

All NGS reads were mapped to the hg19 reference genome using bowtie2 [31] and ATAC-seq and ChIP-seq peaks were identified using MACS2 using default parameters [32]. Differentially enriched sites and expressed genes were identified using DESeq2 [33]. sgRNA enrichments were determined using DESeq2 [33] and aggregate sgRNA log2 fold changes were calculated using MAGeCK [34]. Additional details are provided in Supplementary Methods.

## RESULTS

### Glucocorticoid response leads to pervasive effects on the ALL chromatin landscape

The cellular response to GCs was mapped in two human B-ALL cell lines (697 and Nalm6) using diverse functional genomic assays over a time course spanning a 24 h window (0, 2, 6, 12, and 24 h timepoints; schematic in Supplementary Fig. 1) following treatment with prednisolone [13]. Importantly, cell viability was not affected during the time course.

**Fig. 1:**
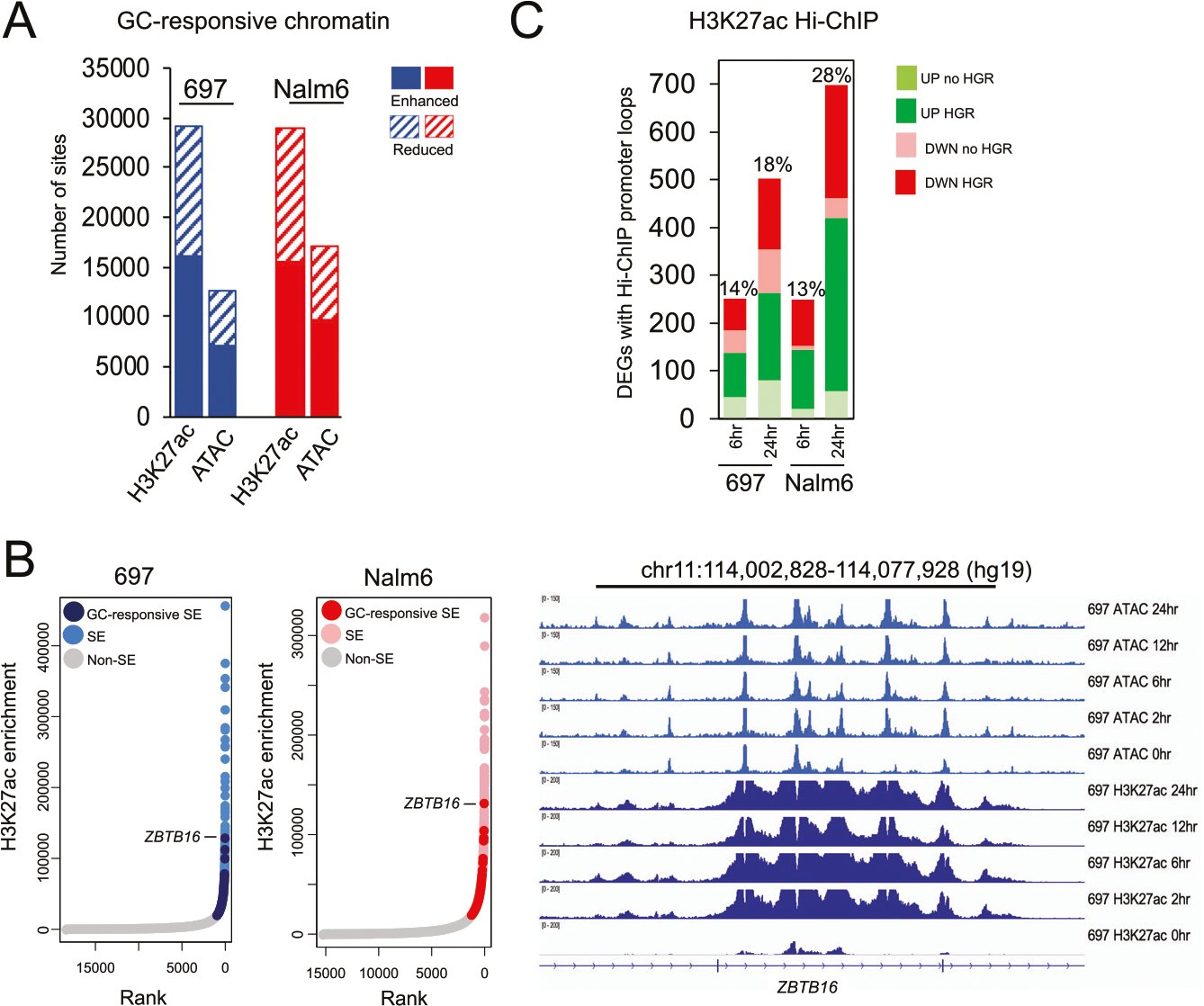
Epigenomic response to glucocorticoids. **A** Number of GC-responsive changes to H3K27ac enrichment and accessible chromatin (ATAC-seq; ATAC) in 697 (blue) and Nalm6 (red) cells across all timepoints is provided. Enhanced and reduced GC-responsive changes in chromatin state are shown separately. **B** H3K27ac enrichment ranking of SEs in 697 (left) and Nalm6 (right) cells is shown at the left. GC-responsive SEs are shown in dark blue (697) and red (Nalm6), pre-established or baseline SEs are shown in light blue (697) and pink (Nalm6) and non-SEs are shown in gray. The intragenic *ZBTB16* SE is marked. IGV browser tracks of 697 ATAC-seq and H3K27ac enrichment at the intragenic *ZBTB16* SE is provided at the right. **C** Number of H3K27ac HiChIP promoter loops to DEGs (absolute fold change >2) in 697 and Nalm6 cells, and after 6 and 24 h of GC treatment is shown. Upregulated DEGs are shown in green and downregulated DEGs are shown in red. Number of HiChIP promoter loops involving HGRs (green or red) and not involving HGRs (light green and pink) are provided. The percentage of total DEGs at each timepoint with HiChIP loops is provided above each plot.

We assessed for GC-responsive epigenomic changes using ATAC-seq and ChIP-seq for the histone H3 lysine 27 acetylation (H3K27ac) post-translational modification, both of which mark active *cis*-regulatory elements [35–37]. In total, 12597 and 16984 changes to chromatin accessibility, as well as 29073 and 28854 alterations in H3K27ac enrichment were uncovered in 697 and Nalm6 cell lines, respectively across all timepoints (FDR < 0.01; Fig. 1A). These two chromatin features also demonstrated opposing temporal patterns, with most changes to acetylation occurring early within 6 h of GC treatment, whereas most accessibility changes occurred after 6 h (Supplementary Fig. 2). Notably, the extent of GC-responsive chromatin alterations was in line with previous investigations in the non-leukemic A549 cell line [38] (Supplementary Fig. 3). However, we identified substantial cell type-specificity, with only 5.8% (ATAC-seq) and 8.3% (H3K27ac) of GC-responsive sites shared, on average, between leukemic and non-leukemic cell lines, which is consistent with our previous findings demonstrating low nuclear receptor binding concordance between distinct cell types [39].

Because GC treatment leads to changes in chromatin state, we wanted to further determine if GCs promote the formation of super-enhancers (SEs). Rank Ordering of Super-Enhancers (ROSE) [40, 41] using H3K27ac data identified 278 (697) and 254 (Nalm6) reproducible GC-responsive SEs following GC treatment present at two or more timepoints. The vast majority were *de novo* SEs that were identified after GC treatment, whereas a small subset was pre-established SEs showing >2-fold H3K27ac enrichment following GC treatment (Supplementary Fig. 4). Notably, the top GC-responsive SE in each cell line was located within the *ZBTB16* gene locus, a GC response gene identified in vivo [42] (Fig. 1B).

Glucocorticoid receptor (GR) ChIP-seq over the time course identified that 54.9% (697) and 58.1% (Nalm6) GR occupancy sites harbored a discernable GR motif, consistent with previous studies [39] (Supplementary Fig. 5). Notably, most GC-responsive accessible chromatin (61%) and H3K27ac (52%) sites harbored GR occupancy (Supplementary Fig. 6), including all GC-responsive SEs. To identify high-confidence sites of GR occupancy mapping to *cis-*regulatory elements, we intersected GR sites with ATAC-seq and H3K27ac peaks at each timepoint (GR + ATAC-seq + H3K27ac sites; henceforth referred to as HGRs) and uncovered 26096 and 29058 HGRs in 697 and Nalm6 cells, respectively. Motif analyses uncovered that HGRs with canonical GRE motifs preferentially harbor enhanced chromatin accessibility and H3K27ac enrichment following GC treatment compared to HGR without GREs (Fisher’s Exact *p* < 2.2 × 10^−16^; Supplementary Fig. 7), consistent with a role in transcriptional activation.

RNA-seq (0, 6, and 24 h) identified 3414 and 2767 differentially expressed genes (DEGs, FDR <0.01, absolute fold change >2; combined DEGs from 6 and 24 h) in 697 and Nalm6 cells, respectively. Supporting a role for GR in regulating the expression of many of these GC-responsive genes, HGRs with enhanced H3K27ac following GC treatment were enriched near upregulated genes (K-S test p<2.2 × 10^−16^; Supplementary Figs. 8, 9). By contrast, HGRs with reduced H3K27ac following GC treatment were enriched near downregulated genes (K-S test *p* < 7.9 × 10^−10^). To more directly link HGRs and GC-responsive chromatin alterations with transcriptional effects we performed H3K27ac HiChIP [43] to map long-range, three-dimensional interactions between distal *cis*-regulatory elements and promoters (0, 6, and 24 h). Most loops (FitHiChIP q < 0.01) were identified after 24 h of GC treatment, and an average of 37.8% (697) and 31.7% (Nalm6) of all loops were interactions between distal *cis*-regulatory elements and promoters (Supplementary Fig. 10). Notably, 14% and 18% (697) as well as 13% and 28% (Nalm6) of 6 and 24 h HiChIP promoter loops mapped to DEGs (absolute fold change >2) at 6 and 24 h respectively, and 65% (697) and 88% (Nalm6) of these loops, on average, involved HGRs (Fig. 1C). GC-responsive H3K27ac alterations further correlated with HiChIP promoter looping at GC-responsive genes. For all loops to DEG promoters (absolute fold change >2), GC-responsive chromatin sites exhibiting enhanced H3K27ac preferentially looped to upregulated gene promoters compared to downregulated gene promoters (697 *p* < 2.2 × 10^−16^, 6 hr DEG odds ratio = 10, 24 h DEG odds ratio = 5.2; Nalm6 *p* < 8.6 × 10^−8^, 6 h DEG odds ratio = 2.3, 24 h DEG odds ratio = 4.7; Fisher’s Exact), while GC-responsive chromatin sites exhibiting reduced H3K27ac preferentially looped to downregulated gene promoters compared to upregulated gene promoters (697 *p* < 2.9 × 10^−11^, 6 h DEG odds ratio = 6.3, 24 h DEG odds ratio = 7.2; Nalm6 *p* <2.2 × 10^−16^, 6 h DEG odds ratio = 13.8, 24 h DEG odds ratio = 7.1; Fisher’s Exact). Taken together, these data indicate that GCs elicit pervasive changes to chromatin state, including the formation of GC-responsive SEs, and these chromatin alterations drive transcriptional programs in ALL cell lines through long-range looping at sites of GR occupancy.

### Identification of transcription factors impacted by GC responses

**Fig. 2:**
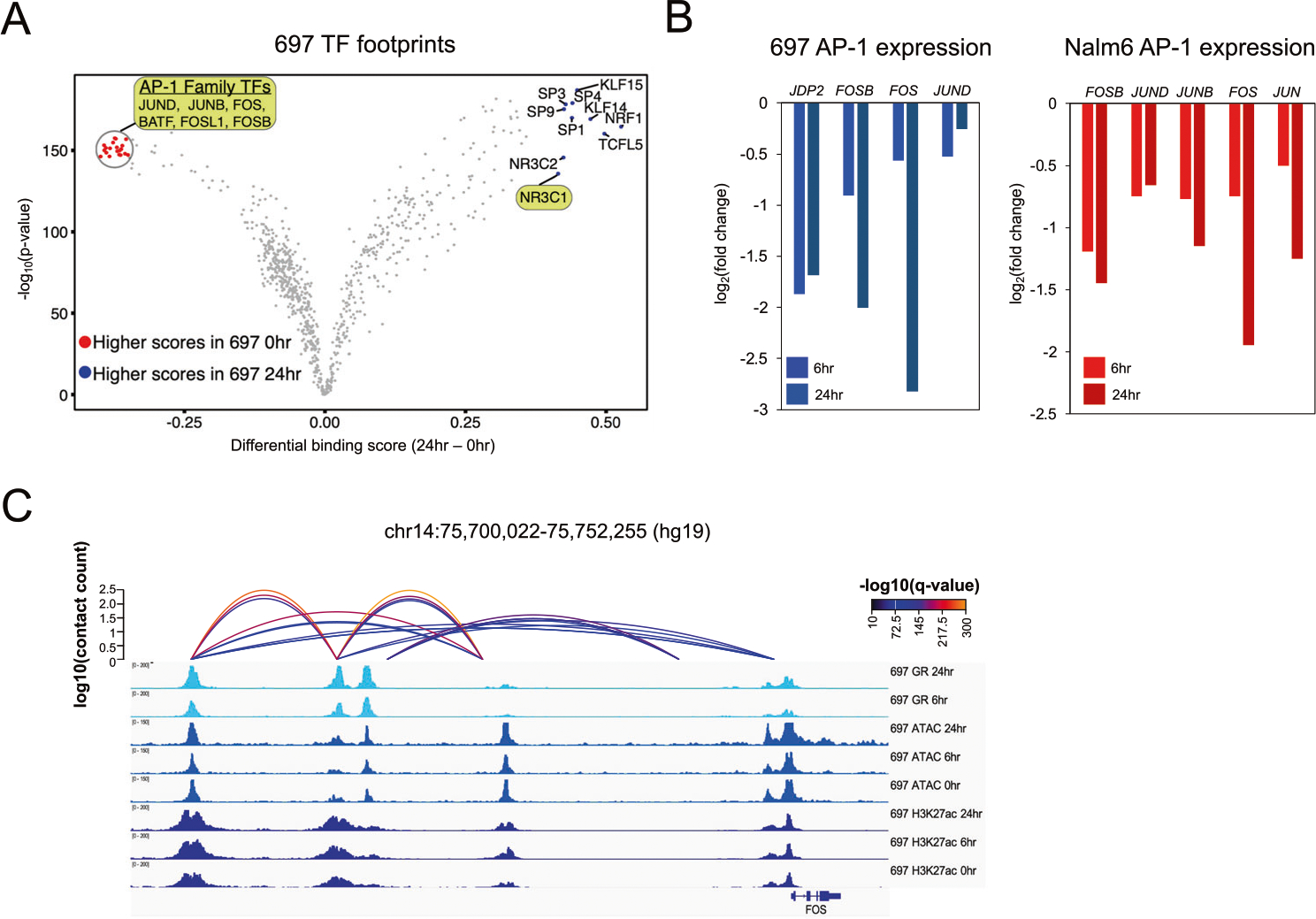
GC-responsive repression of AP-1 transcription factors. **A** TOBIAS TF footprint score differences (x-axis) and their significance (y-axis) between 0 and 24 h timepoints in 697 cell lines is shown. Significant AP-1 family TF footprints with stronger scores at 0 h are highlighted and denoted in red. Several outlier TFs with significantly stronger scores at 24 h are depicted in blue, including GR or NR3C1 which is also highlighted. **B** RNA-seq log2 fold change of AP-1 TFs that are significantly repressed following 6 and 24 h of GC treatment in 697 (left) and Nalm6 (right) cells. **C** IGV browser tracks of HiChIP loops between a distal HGR and *FOS* promoter in 697 cells.

Transcription factor (TF) footprints [44] at accessible chromatin sites harboring GR occupancy were integrated with RNA-seq to identify candidate TFs that cooperated with GR and identified numerous ETS-family TFs (Supplementary Figs. 11–13). TF footprints were further used to identify GC-induced changes in TF occupancy (Fig. 2A, Supplementary Figs. 14, 15). As expected, the GR motif (represented by NR3C1) was enriched following GC treatment. However, a strong depletion of AP-1 footprints was also observed. Concordant with these findings, accessible chromatin sites with AP-1 footprints were significantly enriched at GC-responsive sites with reduced accessibility following GC treatment compared to accessible chromatin sites devoid of AP-1 footprints (Fisher’s Exact *p* < 2.2 × 10^−16^, 697 odds ratio = 5.8, Nalm6 odds ratio = 5). Multiple AP-1 TFs were also downregulated following GC treatment (Fig. 2B), and HiChIP loops were uncovered between distal HGRs and the promoters of *FOS, FOSB* and *JDP2* (697) or *FOSB* and *JUN* (Nalm6), supporting a direct role for GR in AP-1 transcriptional repression (Fig. 2C).

To explore these data further, PECA statistical analysis [45] using chromatin accessibility and gene expression was used to infer changes in TF-gene connections after GC treatment. Validating our findings, GR showed enhanced gene connectivity (Supplementary Fig. 16), and numerous AP-1 TFs exhibited reduced connectivity. Moreover, ETS-family TFs exhibited enhanced connectivity, supporting their role as GR cooperating TFs. Using GREAT [46], we further determined that GC-responsive AP-1 bound sites were associated with genes involved in cell proliferation and anti-apoptotic processes (Supplementary Fig. 17), and concordant pathways were uncovered when limiting associated genes to downregulated genes (fold change <2 or <1.5). Collectively these data suggest that the repression of AP-1-bound *cis*-regulatory elements and genes precedes GC-induced apoptosis

### Identification of transcriptionally active sequences using ATAC-STARR-seq

**Fig. 3:**
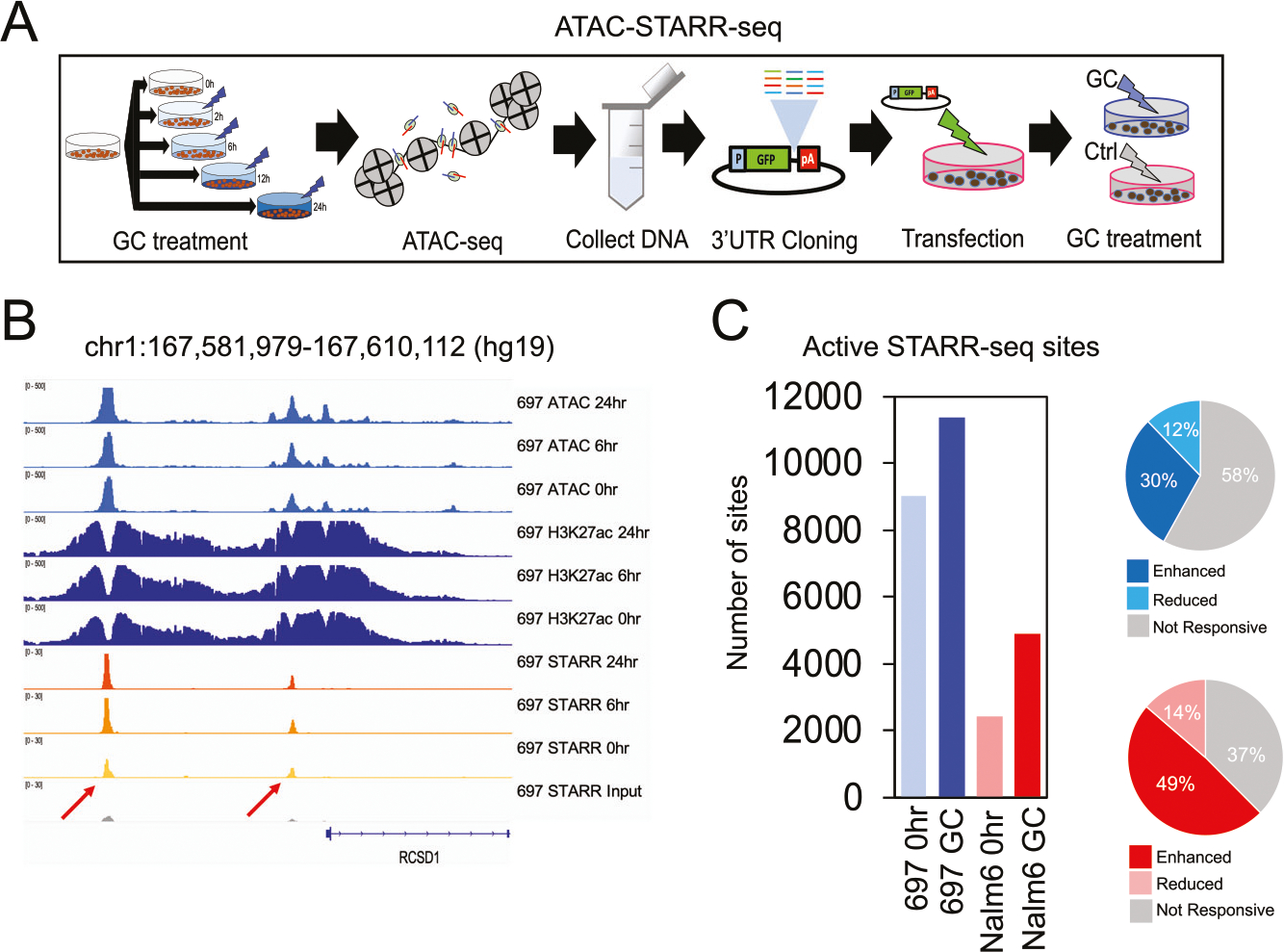
Functional validation of GC-responsive sites using high-throughput reporter assays. **A** A schematic of diagram of ATAC-STARR-seq is provided. **B** IGV browser read count tracks of 697 ATAC-seq and H3K27ac, as well as read counts per million tracks of ATAC-STARR-seq DNA input and RNA output in 697 cells at 0, 6, and 24 h are provided. Examples of two active STARR-seq sites in 697 cells near the *RCSD1* GC-responsive gene are denoted by red arrows. **C** Number of active STARR-seq sites identified after 0 h or after 6 + 24 h (GC) of prednisolone treatment in 697 and Nalm6 cells is provided at the left. Percentage of GC-active STARR-seq sites in 697 (top) and Nalm6 (bottom) cells that map to GC-responsive ATAC-seq sites exhibiting enhanced (blue and red) or reduced (light blue and pink) open chromatin accessibility following GC treatment is provided at the right.

Self-transcribing active regulatory region sequencing [47, 48] at accessible chromatin sites (ATAC-STARR-seq) were performed in 697 and Nalm6 cell lines to functionally validate the activity of GC-responsive chromatin sites and HGRs (Fig. 3A). However, this assay can identify activity for all cloned ATAC-seq accessible chromatin sites, irrespective if they are occupied by GR or GC-responsive. To control for effects from variable DNA sequence length, ATAC-seq transposed DNA was size selected prior to cloning into the STARR-seq plasmid. As validation of our plasmid library, we determined that 98% (697) and 97% (Nalm6) of accessible chromatin sites were cloned into plasmids (Supplementary Fig. 18). Cellular transfections in each ALL cell line were performed using 4 bulk transfections that were subsequently split to create the cell populations for the 3 different treatment conditions (GCs for 0, 6, or 24 h; *n* = 4 replicates per treatment condition in each cell line) to control for variation in transfection efficiencies between treatment conditions. Following transfection of ATAC-STARR-seq plasmid libraries and GC treatment (for 0, 6, or 24 h), RNA output libraries from replicate treatment conditions were sequenced and merged to increase total sequence depth and coverage. RNA output libraries were also downsampled to control for read count differences between treatment conditions and active STARR-seq sites at 0, 6, and 24 h were identified by significant increases in RNA output versus DNA input using BasicSTARRseq (https://git.bioconductor.org/packages/BasicSTARRseq).

On average, we identified 8864 (697) and 3101 (Nalm6) active STARR-seq sites (*p* < 0.001) at each timepoint (examples in Fig. 2B). As additional verification of these findings, we implemented a second approach to identify active STARR-seq sites using DESeq2 [33] differential analysis that compared enrichment at accessible chromatin sites of ATAC-STARR-seq RNA output replicates (*n* = 4) over ATAC-seq pooled input replicates (*n* = 3) that consisted of ATAC-seq replicates (#1, 2 or 3) pooled across the time course (e.g., pooled replicate #1 for 0, 2, 6, 12, and 24 h, etc.; Supplementary Methods). Supporting this approach, ATAC-STARR-seq plasmid DNA input was highly correlated with pooled ATAC-seq signal (697 *r*^*2*^ = 0.95, Nalm6 *r*^*2*^ = 0.94; Supplementary Fig. 19). Consistently, nearly all genomic regions identified as active STARR-seq sites using the DESeq2-based approach (FDR < 0.05) were also identified by the BasicSTARRseq approach (697 = 95.9%, Nalm6 = 88.4%), validating our BasicSTARRseq findings and supporting the overall reproducibility and robustness of our ATAC-STARR-seq results.

Because active STARR-seq sites after 6 and 24 h of GC treatment showed substantial overlap and read count correlation (Supplementary Fig. 20), they were combined for all downstream analyses (henceforth named GC-active STARR-seq sites; Fig. 3C). Over 66% (697) or 83% (Nalm6) of GC-active STARR-seq sites mapped to HGRs. Moreover, 697 and Nalm6 GC-responsive open chromatin sites were significantly enriched at GC-active STARR-seq sites compared to accessible chromatin sites that were not GC-responsive (Fisher’s Exact *p* < 5.4 × 10^10^; Fig. 3C, Supplementary Fig. 21). Notably, GC-responsive chromatin sites exhibiting enhanced accessibility were significantly more enriched at GC-active STARR-seq sites compared to sites showing reduced accessibility (Chi-square *p* < 2.2 × 10^−16^; 2.2–3.5-fold).

We further compared active STARR-seq sites before (0 h) and after (GC) GC treatment to identify sites that are active only after GC exposure (i.e., GC-specific active STARR-seq site). Shared active STARR-seq sites between 0 h and GC conditions exhibited substantially greater overlap with promoter locations compared to 0 h-specific and GC-specific active STARR-seq sites, which had a higher proportion of promoter-distal elements (Supplementary Fig. 22). Importantly, GREs were significantly more enriched at GC-specific active STARR-seq sites in 697 cells compared to 0 h-specific active STARR-seq sites (Chi-square *p* = 1.3 × 10^−4^, 1.5-fold) and exhibited a strong trend for greater enrichment in Nalm6 cells (Chi-square *p* = 0.077, 1.6-fold). GC-specific active STARR-seq sites were also more closely associated with GC-responsive upregulated genes compared to 0 h-specific active STARR-seq sites (24 h DEGs, fold change >2; K-S test *p* = 0.05 [697] and *p* = 0.002 [Nalm6]). Overall, ATAC-STARR-seq validated gene regulatory activity for thousands of GC-responsive accessible chromatin sites, and most harbor GR occupancy.

### Genetic disruptions to the GC response impact GC resistance in patient samples

Resistance of primary ALL cells to GCs is predictive of treatment response in patients measured as either persistence of minimal residual disease after remission induction treatment or overall treatment outcome [4–6, 10, 49, 50]. Thus, ex vivo measurements of primary ALL cell resistance to GCs is concordant with in vivo resistance and predicts treatment outcome in patients. We therefore investigated the impact of inherited genetic variants at HGRs that were associated with ex vivo GC resistance in primary cells.

**Fig. 4:**
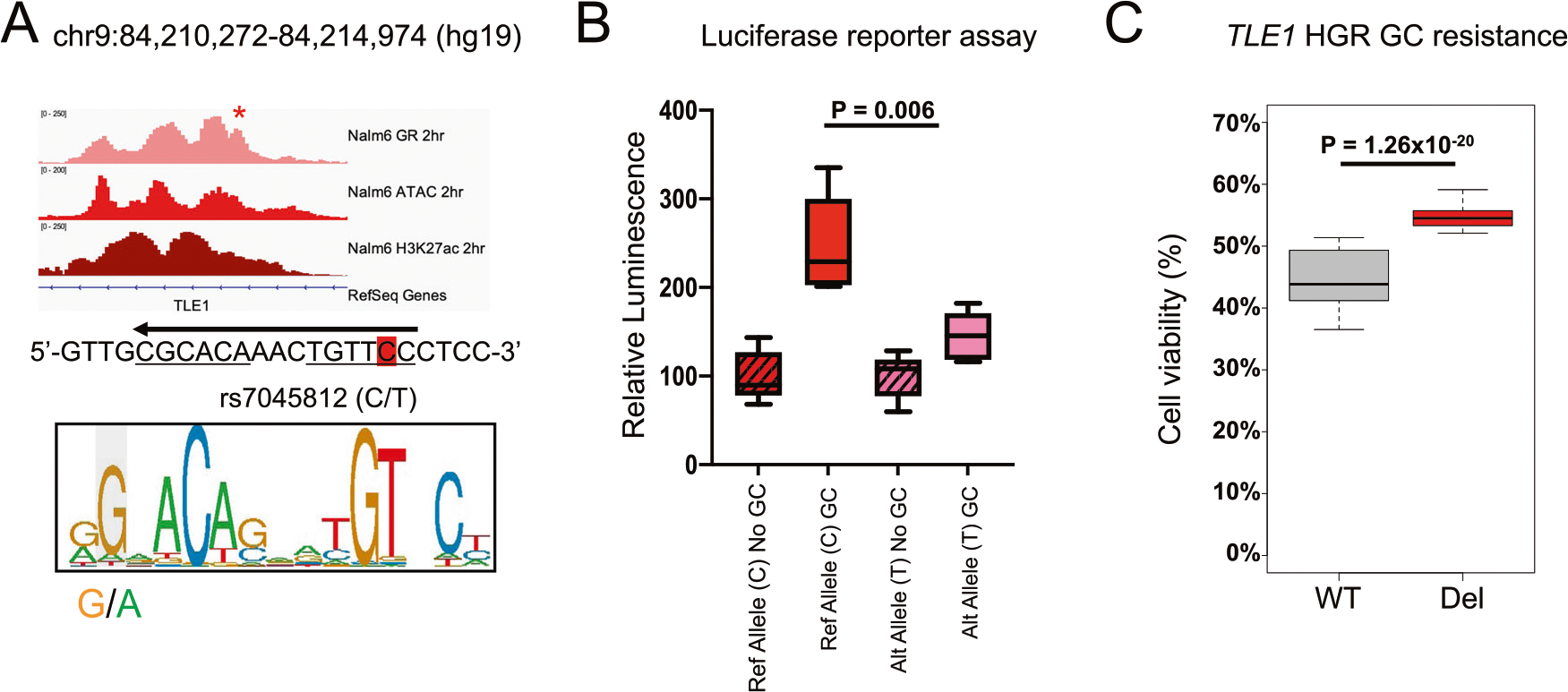
Impact of intragenic variant rs7045812 on GC-responsiveness and *TLE1* expression. **A** IGV browser tracks of Nalm6 GR, ATAC-seq and H3K27ac at HGR spanning variant rs7045812 after 2 h of GC treatment is shown. The genome sequence spanning variant rs7045812 (highlighted in red) is shown and the GRE sequence (negative strand; arrow) is underlined. The GRE motif (positive strand) is shown below and the location of rs7045812 is denoted. **B** Luciferase reporter assay testing a 300-bp fragment of DNA centered on the reference C allele and alternative T allele in the presence or absence of prednisolone (5 μM; *n* = 5 per group). **C** GC drug viability results displaying the percentage of viable cells after 72 h of prednisolone treatment in parental/wild-type (Wt) and *TLE1* HGR deleted (Del) Nalm6 cells (250 nM; *n* = 48 per group).

Using previously published genotyping and ex vivo GC drug sensitivity data in primary ALL cells from patients enrolled in St. Jude Total Therapy XVI [17, 18], we intersected variants associated with resistance to prednisolone and/or dexamethasone and variants in high linkage disequilibrium (r^2^ > 0.8) with HGRs in 697 and Nalm6 cell lines and fine-mapped 45 variants to HGRs (Supplementary Table 1), including several that were expression quantitative trait loci (eQTLs) for six genes previously implicated in GC resistance *(ARHGAP18, ATG10, BFSP2, PPM1E, TLE1*, and *XRRA1)* [18]. A notable variant was rs7045812 (C/T) which mapped to a Nalm6 HGR and altered a GRE (Fig. 4A). This intragenic variant is located within the *TLE1* gene locus, a GC-responsive upregulated gene that has also been previously correlated with prognostic features in ALL patients [51] and functions with Groucho as a canonical Wnt signaling repressor [52, 53]. In line with the recognized consensus GRE, the alternative T allele, which disrupts the GRE, is associated with greater GC resistance in primary cells (Supplementary Fig. 23). In support of these data, luciferase reporter assays testing 300-bp DNA fragments centered on rs7045812 confirmed that the alternative T allele negatively impacted GC-responsiveness compared to the reference C allele (Fig. 4B, Supplementary Fig. 24). CRISPR/Cas9 genome editing was further used to delete the *TLE1* HGR in Nalm6 cell lines and resulted in greater GC resistance (Fig. 4C, Supplementary Fig. 25).

Notably, HiChIP identified long-range promoter interactions between rs7045812 and the *TLE1* promoter (Supplementary Fig. 26). In line with this observation, *TLE1* expression was significantly reduced in *TLE1* HGR deleted cells compared to wild-type cells, thereby validating the gene regulatory effects of this HGR on *TLE1* expression (Supplementary Fig. 27). We further tested the impact of *TLE1* disruption on GC resistance using CRISPR/Cas9 knockout in Nalm6 cell lines (Supplementary Fig. 28). GC drug response assays using prednisolone uncovered that *TLE1* disruption led to greater GC resistance (Supplementary Fig. 29), and this was concordant with both lower *TLE1* expression being associated with GC resistance in primary cells (Supplementary Fig. 30) and with greater GC resistance in *TLE1* HGR deleted cells (Fig. 4C). Collectively, these data highlight a role for inherited genetic variation at sites of GR occupancy in GC resistance.

### Integrative analysis identifies epigenetic disruptions to the GC response impacting GC resistance in patient samples

**Fig. 5:**
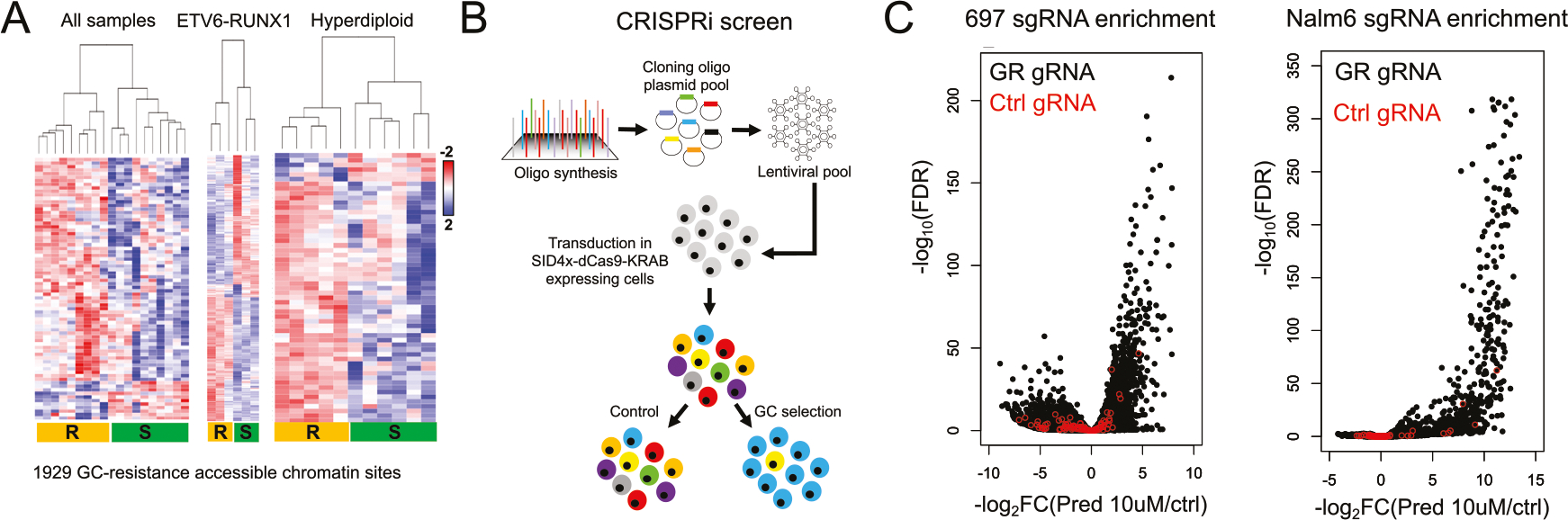
CRISPRi screen of GR-occupied chromatin accessibility sites associated with GC resistance. **A** Heatmap of accessible chromatin sites associated with GC resistance. Heatmaps of analyses using all patient samples as well as intra-subtype analyses (ETV6-RUNX1 and Hyperdiploid) are provided. **B** A schematic of the CRISPR interference (CRISPRi) screen is provided. **C** Volcano plot of individual sgRNA log_2_ fold changes (x-axis) and significance (y-axis, log_10_ FDR) following prednisolone treatment in 697 (left) and Nalm6 (right) cells is provided. Control, non-targeting gRNAs are depicted in red.

Epigenetic disruptions to HGRs were also explored to better understand their impact on GC resistance. Using published ATAC-seq data in primary ALL cells from our laboratory [26], ex vivo GC drug sensitivity assays were performed on 19 of these primary ALL cells. We stratified cells by GC resistance (Supplementary Fig. 31) and identified 1929 sites where differential chromatin accessibility was associated with GC resistance (FDR < 0.1) through inter-subtype (all samples) and/or intra-subtype (only ETV6-RUNX1 or hyperdiploid samples) analysis (Fig. 5A, Supplementary Table 2; henceforth referred to as GC-resistance accessible chromatin sites). To understand factors contributing to chromatin accessibility differences we examined DNA methylation that was available for a subset of patient samples. Of the 908 GC-resistance accessible chromatin sites that overlapped with 2566 CpG probes, 85% of probes exhibited patterns consistent with chromatin accessibility differences between GC-sensitive and GC-resistant samples, highlighting DNA methylation as a contributor to chromatin alterations (Supplementary Fig. 32). We also uncovered that GC-resistance accessible chromatin sites were significantly enriched at HGRs compared to accessible chromatin sites not associated with GC-resistance (Fisher’s Exact *p* < 2.2 × 10^−16^, odds ratio = 1.73). Most of these GC-resistance accessible chromatin sites (78%) exhibited greater occlusion in GC-resistant primary cells, concordant with the role of GCs as a chemotherapeutic drug promoting apoptosis (Supplementary Fig. 33). To identify top candidate HGRs impacting GC resistance in patient samples, we performed an integrative analysis that combined GC-resistance accessible chromatin sites with GC response maps, genes implicated in GC resistance [18] and CRISPR interference (CRISPRi) screening (Supplementary Fig. 34).

CRISPRi using Enhancer-i [54] was used to screen all GR occupancy sites at GC-resistance accessible chromatin sites for GC resistance phenotypes using 11038 sgRNAs and 100 non-targeting control sgRNAs (Fig. 5B and Supplementary Table 3). Following a 72 h drug selection with prednisolone, we identified numerous sgRNAs exhibiting significant enrichment (697 = 1844, Nalm6 = 774; FDR < 0.05; Supplementary Tables 4, 5), whereas control sgRNAs overall did not show strong or preferential enrichment (Fig. 5C). By further aggregating all sgRNA data at each GR site using MAGeCK [34] (~6.5 sgRNAs per site), we ranked GR sites by log_2_-transformed fold change enrichment (Supplementary Tables 6, 7). As expected, control sgRNAs were situated near the center of the ranking and did not exhibit strong sgRNA enrichment or depletion (Supplementary Fig. 35). We next identified CRISPRi-enriched GR sites that mapped to HGRs (log_2_ fold change >0), determined if these HGRs were associated with GC-responsive genes using GREAT [46] and examined if these genes had been implicated in GC resistance [18]. This integrative analysis identified 35 top HGRs associated with 26 GC-responsive genes implicated in GC resistance (Supplementary Table 8).

### Functional evaluation of epigenetically disrupted HGRs

**Fig. 6:**
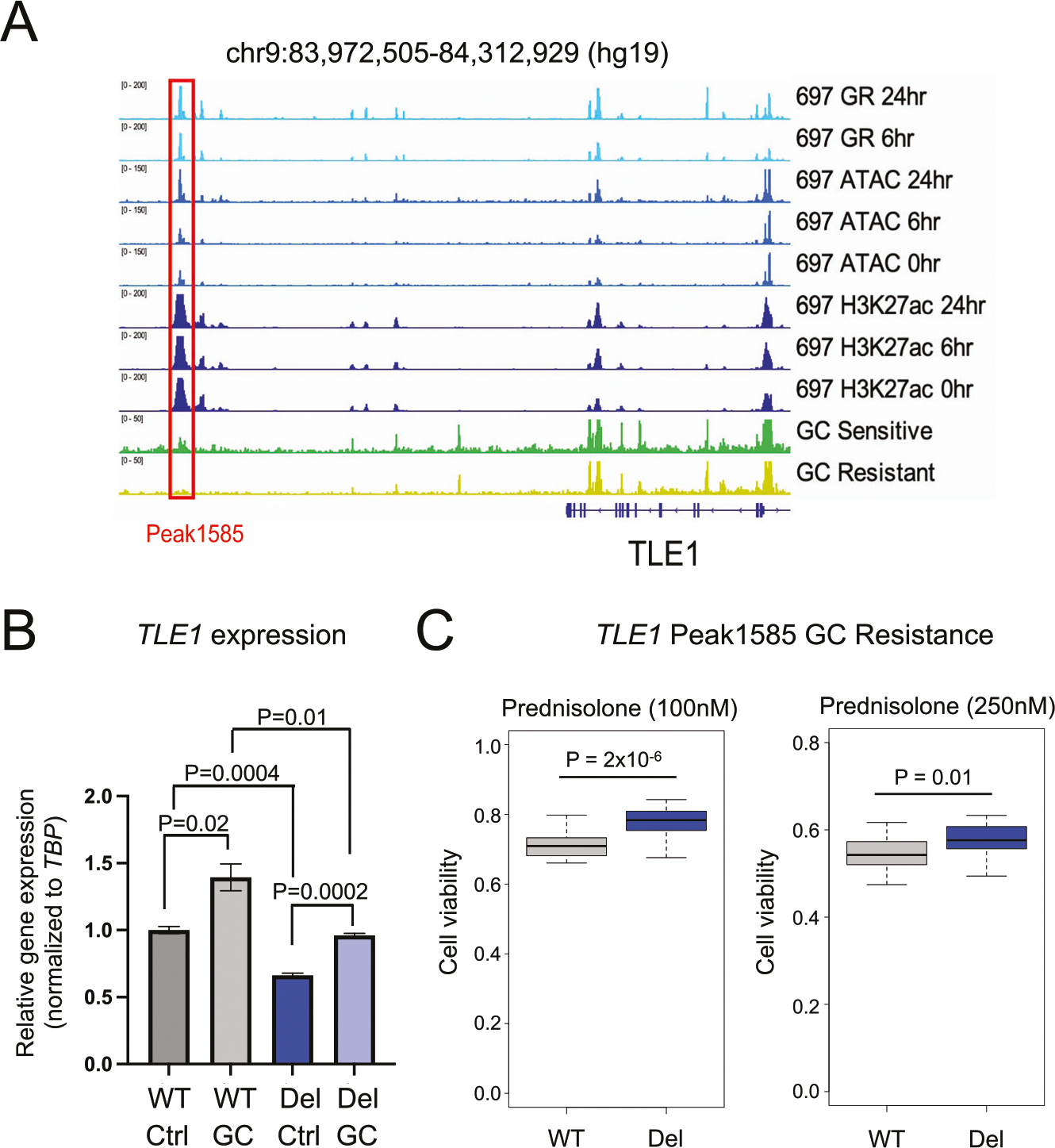
Functional evaluation of an epigenetically disrupted HGR at the *TLE1* gene locus. **A** IGV browser tracks of 697 GR, ATAC-seq and H3K27ac is provided near the *TLE1* gene locus along with representative ATAC-seq for GC-sensitive and GC-resistant ETV6-RUNX1 primary ALL cell samples from patients. Tracks denote reads counts. A CRISPRi-enriched HGR Peak1585 is outlined in red. **B** *TLEI* RT-qPCR results of 697 parental/wild-type (WT) and HGR Peak1585 deleted (Del) 697 cells is shown in the presence (GC) and absence (Ctrl) of prednisolone (10 μM; *n* = 6 per group). **C** GC drug viability results displaying the fraction of viable cells after 72 h of 100 nM prednisolone treatment (left) or 250 nM prednisolone treatment (right) in parental/wild-type (WT) and *TLE1* Peak1585 deleted (Del) 697 cells (*n* = 24 per group).

Closer functional examinations of top HGRs were performed to further associate epigenetic *cis*-regulatory disruptions in patient samples with GC resistance. We identified a top HGR (Peak1585) in 697 cell lines that was situated downstream of *TLE1* (Fig. 6A). Because we identified *TLE1* as a GC-resistance gene harboring an intragenic HGR variant associated with GC resistance (Fig. 4), we functionally investigated this distal HGR. Greater chromatin occlusion was observed in GC-resistant primary cells, and this was supported by greater CpG DNA methylation in GC-resistant samples (Supplementary Fig. 36). CRISPR/Cas9-mediated deletion of this HGR significantly reduced *TLE1* expression (Fig. 6B, Supplementary Fig. 37) and led to greater GC resistance (Fig. 6C), supporting the functional role of this distal HGR in *TLE1* gene regulation and GC resistance.

**Fig. 7:**
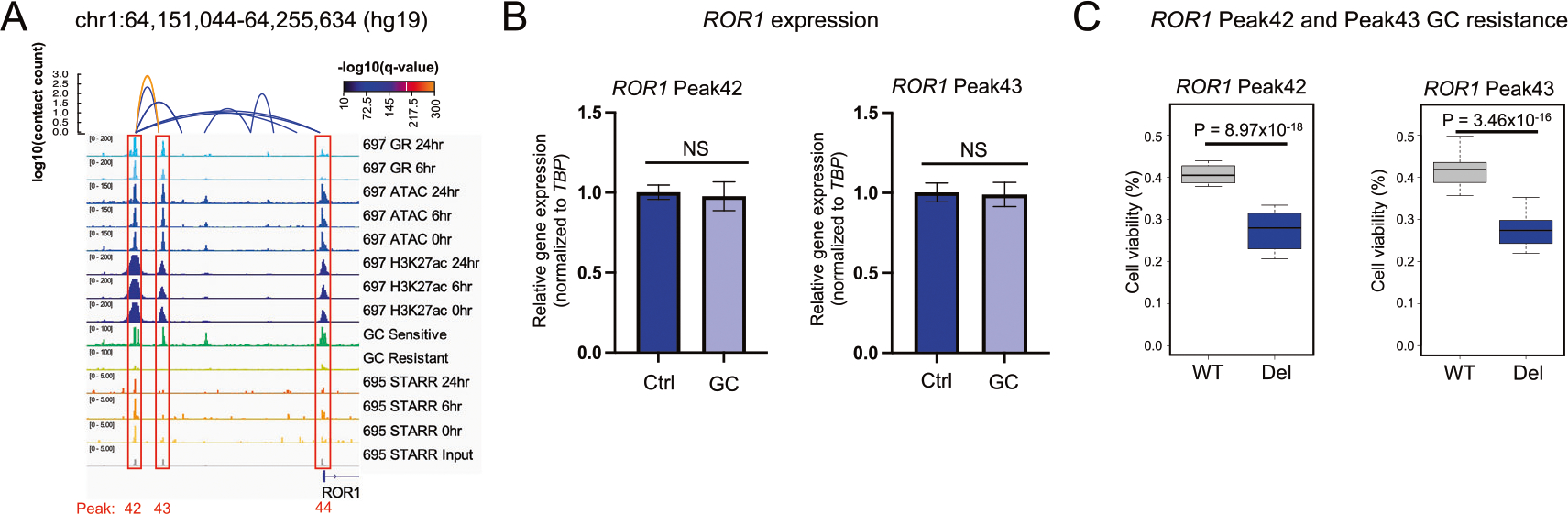
Functional evaluation of epigenetically disrupted HGRs at the *RORI* gene locus. **A** IGV browser tracks of 697 HiChIP loops, GR, ATAC-seq, H3K27ac and ATAC-STARR-seq is provided near the *ROR1* gene locus along with representative ATAC-seq for GC-sensitive and GC-resistant ETV6-RUNX1 primary ALL cell samples from patients. ATAC-STARR-seq tracks are shown as read counts per million and the remaining tracks denote reads counts. Three CRISPRi-enriched HGRs (Peak42, Peak43 and Peak44) are outlined in red and H3K27ac HiChIP loops between Peak42 and Peak43, and between Peak42 and promoter Peak 44 are shown. **B** *ROR1* RT-qPCR results of parental/wild-type (WT) and HGR Peak42 deleted (Del) 697 cells (left) or HGR Peak43 deleted (Del) 697 cells (right) in the presence (GC) and absence (Ctrl) of prednisolone is shown (10 μM; *n* = 6 per group). **C** GC drug viability results displaying the fraction of viable cells after 72 h of prednisolone treatment in parental/wild-type (WT) and *ROR1* Peak42 deleted (Del) 697 cells (left) or *ROR1* Peak43 deleted (Del) 697 cells (right) (250 nM; *n* = 24 per group).

The *ROR1* locus was identified as the top hit because it contained (i) three top HGRs (Peak42, Peak43 and Peak44), (ii) an HGR (Peak42) exhibiting HiChIP looping to the *ROR1* promoter and a neighboring HGR (Peak43) and (ii) an HGR (Peak42) with STARR-seq activity in 697 cell lines (Fig. 7A). *ROR1* is a receptor tyrosine kinase-like orphan receptor for Wnt5a that can induce activation of noncanonical Wnt signaling [55] and has been associated with survival of TCF3-PBX ALL [56]. However, *ROR1* is also expressed in other ALL molecular subtypes (Supplementary Fig. 38). All three HGRs exhibited greater chromatin occlusion in GC-resistant samples and these alterations were further supported by greater DNA methylation and lower ROR1 expression in GC-resistant samples (Supplementary Figs. 39, 40). In line with these data, lower *ROR1* expression in primary cells was associated with greater GC resistance in an independent patient cohort (Supplementary Fig. 41) and CRISPR/Cas9 disruption of *ROR1* in 697 cell lines led to greater GC resistance (Supplementary Figs. 42, 43). Because *ROR1* is repressed by GCs, GC activity at this gene locus is anti-apoptotic (RNA-seq log_2_ fold change = −0.61, Supplementary Fig. 44). GC-induced *ROR1* repression is also consistent with decreased STARR-seq activity at Peak42 at 24 h and a significant reduction in H3K27ac enrichment at Peak43 after GC treatment (FDR < 0.01). Concordant with these observations, individual CRISPR/Cas9-mediated deletions of the two distal HGRs ablated GC-induced *ROR1* repression and decreased GC resistance (Fig. 7B, C, Supplementary Fig. 45), but baseline *ROR1* expression was not significantly impacted (Supplementary Fig. 46). Overall, these data uncovered that *cis*-regulatory epigenetic disruptions to GC responses are a mechanism impacting GC resistance in ALL.

## DISCUSSION

In this study we map the genome-wide response to GCs in ALL cell lines and identified pervasive effects on the chromatin landscape, including the identification of GC-responsive chromatin sites and SEs, with most harboring GR occupancy. Transcriptomic and three-dimensional looping information further established a role for GC-responsive chromatin sites and HGRs in gene regulation, whereas investigations of TFs involved in the GC response uncovered a repression of AP-1 genes and AP-1 bound *cis*-regulatory elements implicated in cell proliferation and anti-apoptotic processes. We identified a direct role for GR in the repression of AP-1 TFs through HiChIP looping between HGRs and AP-1 promoters, and in the repression of AP-1 *cis*-regulatory elements through direct GR occupancy. To functionally evaluate *cis*-regulatory activities, STARR-seq was employed and validated thousands of GC-responsive chromatin sites, most of which mapped to HGRs.

To determine the functional impact of *cis*-regulatory disruptions to GC responses in drug resistance we integrated our GC response maps with genomic data from patient samples. We identified genetic and/or epigenetic disruptions to HGRs at the *TLE1* and *ROR1* gene loci that impact GC resistance. Importantly, because *TLE1* and *ROR1* are involved in canonical and noncanonical Wnt signaling [52, 53, 55], respectively, these analyses suggest that *cis*-regulatory disruptions to Wnt signaling is a mechanism impacting GC resistance in childhood ALL. However, the effects of GCs on *TLE1* are pro-apoptotic, with greater GC-mediated *TLE1* expression associated with lower GC resistance, whereas the effects of GCs on *ROR1* are anti-apoptotic. *ROR1* is repressed by GCs, and *ROR1* disruption leads to greater GC resistance. Consistently, deletion of the distal *ROR1* HGRs that are utilized for GC-induced repression promotes increased GC sensitivity. Our data further suggest that both distal HGRs appear to be necessary for robust *ROR1* repression, but not for maintaining baseline *ROR1* expression, which may be regulated by additional redundant *cis*-regulatory elements. Collectively, these results suggest that the gene regulatory activities of GCs are not exclusively pro-apoptotic, as has been previously reported [19], and alterations to GC-mediated anti-apoptotic processes also appear to play a role in resistance. Future directions should center on defining molecular mechanisms that link Wnt signaling to GC resistance, as well as follow-up investigations of the top epigenetically altered HGRs and variants we identified in this study (Supplementary Tables 1, 8). In addition, the identification of both genetic and epigenetic alterations at HGRs at the *TLE1* gene locus as well as a correlation between *TLE1* expression and prognostic features in ALL patients [51] all highlight *TLE1* as an important gene for further follow-up studies.

Collectively, our study mapped gene regulatory responses to GCs in ALL cells using orthogonal functional genomic assays and high-throughput reporter assays. Our data further suggest that genetic and epigenetic disruptions to this gene regulatory response impact GC resistance in primary cells from patients.

## Supplementary Material

1833642_Figures

1833642_Sup_Methods

1833642_Sup_Tab_1

1833642_Sup_Tab_2

1833642_Sup_Tab_3

1833642_Sup_Tab_4

1833642_Sup_Tab_6

1833642_Sup_Tab_5

1833642_Sup_Tab_7

1833642_Sup_Tab_8

## Data Availability

All functional genomic data from cell lines have been deposited into the Gene Expression Omnibus (GSE175484).
