# Supplementary material for: Epigenomic profiling of glucocorticoid responses identifies cis-regulatory disruptions impacting steroid resistance in childhood acute lymphoblastic leukemia
Source: Leukemia. Author manuscript; Available in PMC 2022 Oct 4. (PMC9522591; doi:10.1038/s41375-022-01685-z)
Supplement: 1833642_Figures [file NIHMS1833642-supplement-1833642_Figures.pdf]

SUPPLEMENTARY FIGURES

Epigenomic profiling of glucocorticoid responses identifies *cis*-regulatory disruptions impacting steroid resistance in childhood acute lymphoblastic leukemia

Brennan P. Bergeron[1,2,3,§], Jonathan D. Diedrich[1,2,§], Yang Zhang[4], Kelly R. Barnett[1,2], Qian Dong[1,2], Daniel C. Ferguson[1,2], Robert J. Autry[1,2,5], Wenjian Yang[1,2], Baranda S. Hansen[6], Colton Smith[1,2], Kristine R. Crews[1,2], Yiping Fan[7], Ching-Hon Pui[1,5,8], Shondra M. Pruett-Miller[3,6], Mary V. Relling[1,2,3], Jun J. Yang[1,2,3,5], Chunliang Li[3,4], William E. Evans[1,2,3] and Daniel Savic[1,2,3,5,*]

[1] Hematological Malignancies Program and Center for Precision Medicine in Leukemia, St. Jude Children's Research Hospital, Memphis, TN 38105, USA.
[2]Department of Pharmacy and Pharmaceutical Sciences, St. Jude Children's Research Hospital, Memphis, TN 38105, USA.
[3]Graduate School of Biomedical Sciences, St. Jude Children's Research Hospital, Memphis, TN 38105, USA.
[4]Department of Tumor Cell Biology, St. Jude Children's Research Hospital, Memphis, TN 38105, USA.
[5]Integrated Biomedical Sciences Program, University of Tennessee Health Science Center, Memphis, TN 38105, USA.
[6]Department of Cell and Molecular Biology and Center for Advanced Genome Engineering, St. Jude Children's Research Hospital, Memphis, TN 38105, USA.
[7]Department of Computational Biology, St. Jude Children's Research Hospital, Memphis, TN 38105, USA.
[8]Department of Oncology, St. Jude Children's Research Hospital, Memphis, TN 38105, USA.

[§] Equal contributions
[*]Corresponding author

Corresponding Author:

Daniel Savic, Ph.D.
Department of Pharmacy and Pharmaceutical Sciences
Division of Pharmaceutical Sciences
St. Jude Children's Research Hospital
262 Danny Thomas Place, MS: 313
Phone: 901-595-5346
Email: daniel.savic@stjude.org

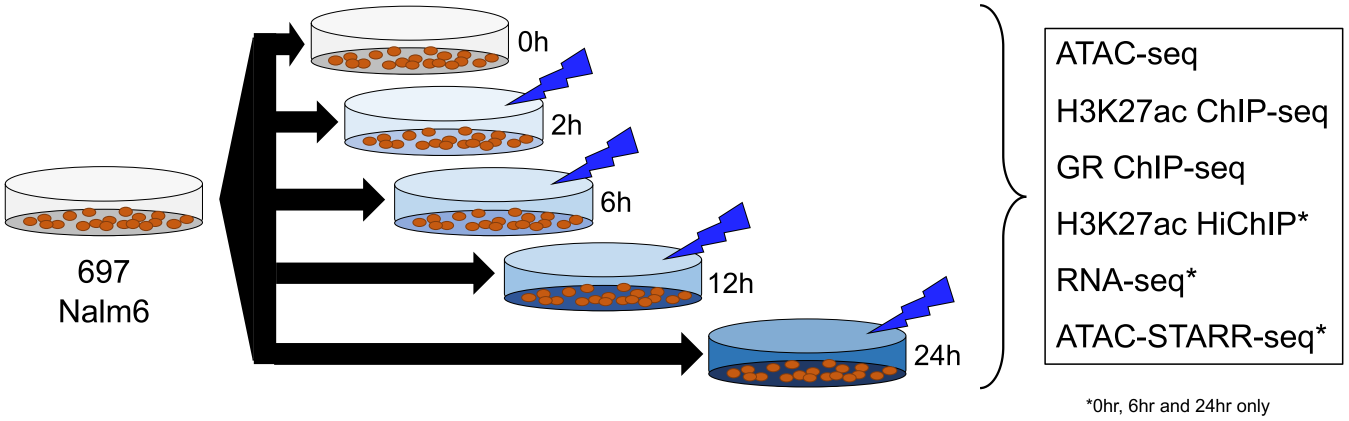

Supplementary Figure 1. Schematic outlining the GC response time course in 697 and Nalm6 cells. ATAC-seq and H3K27ac ChIP-seq were performed to identify changes in chromatin state. GR ChIP-seq was performed to identify sites of GR occupancy. RNA-seq was performed to identify changes in gene expression. H3K27ac HiChIP was used to identify interactions between *cis*-regulatory elements and promoters of differentially expressed genes. ATAC-STARR-seq was used to functionally validate sites of accessible chromatin. H3K27ac HiChIP, RNA-seq and ATAC-STARR-seq were only performed at 0, 6 and 24hrs.

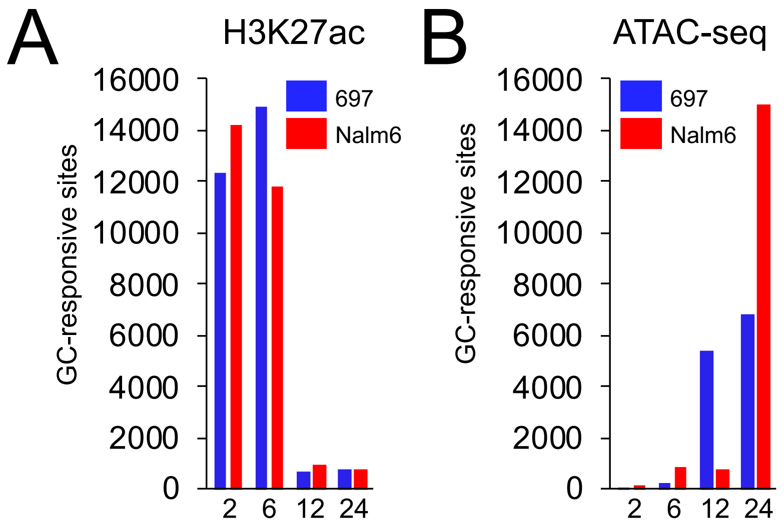

Supplementary Figure 2. (A) Initial GC-responsive changes to H3K27ac enrichment at each site are shown for each timepoint. **(B)** Initial GC-responsive changes to chromatin accessibility at each site are shown for each timepoint.

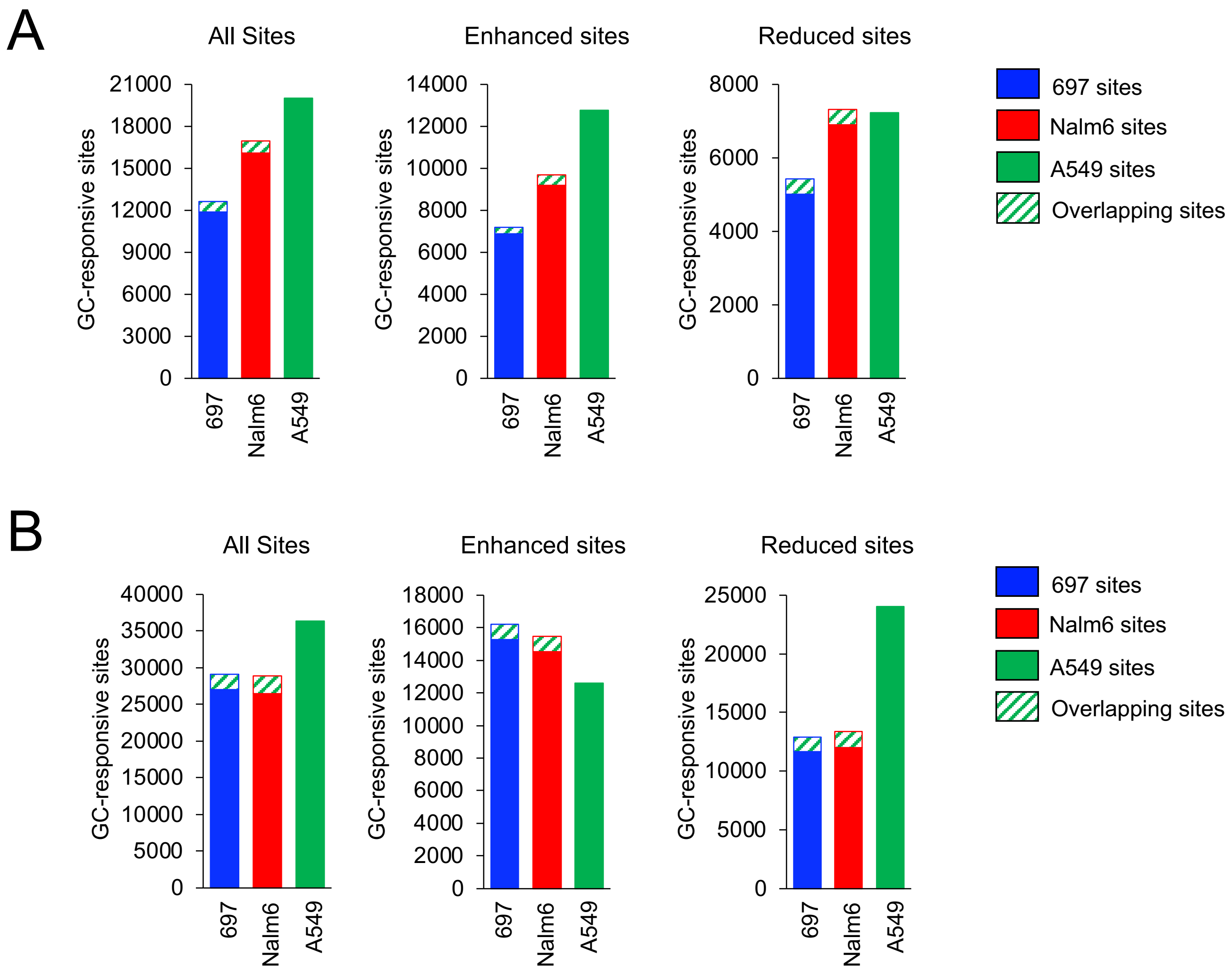

Supplementary Figure 3. Comparisons of GC-responsive ATAC-seq **(A)** and H3K27ac **(B)** sites in 697 (blue), Nalm6 (red) and A549 (green) cell lines. The number of GC-responsive chromatin sites in 697 and Nalm6 cell lines that overlap with GC-responsive sites in the A549 cell line is denoted.

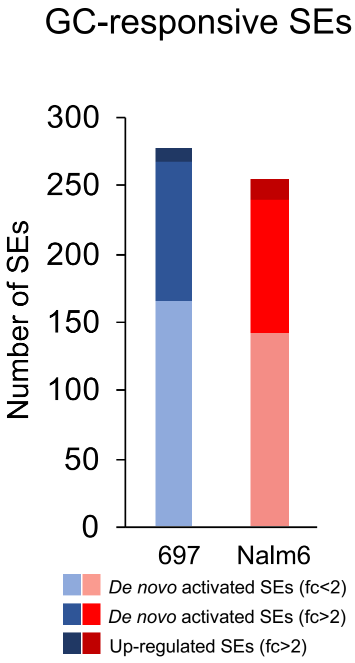

Supplementary Figure 4. Number of GC-responsive super-enhancers (SEs) is provided. The number of *de novo* SEs as well as pre-established SEs showing over 2-fold greater H3K27ac enrichment following GC treatment is provided.

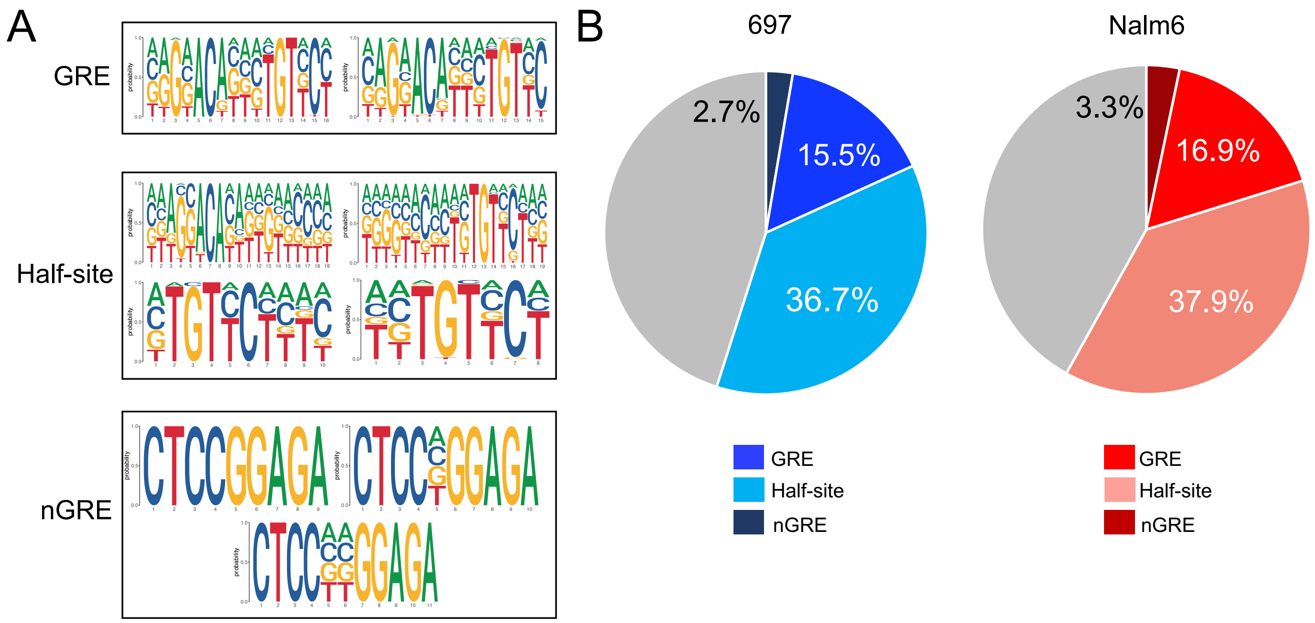

Supplementary Figure 5. (A) Sequence position weight matrices (PWMs) of GREs, weaker half-site motifs and nGREs used to identify discernable GR motifs at GR ChIP-seq sites. **(B)** The percentage of GR ChIP-seq sites harboring these motifs is provided in 697 (left) and Nalm6 (right) cells.

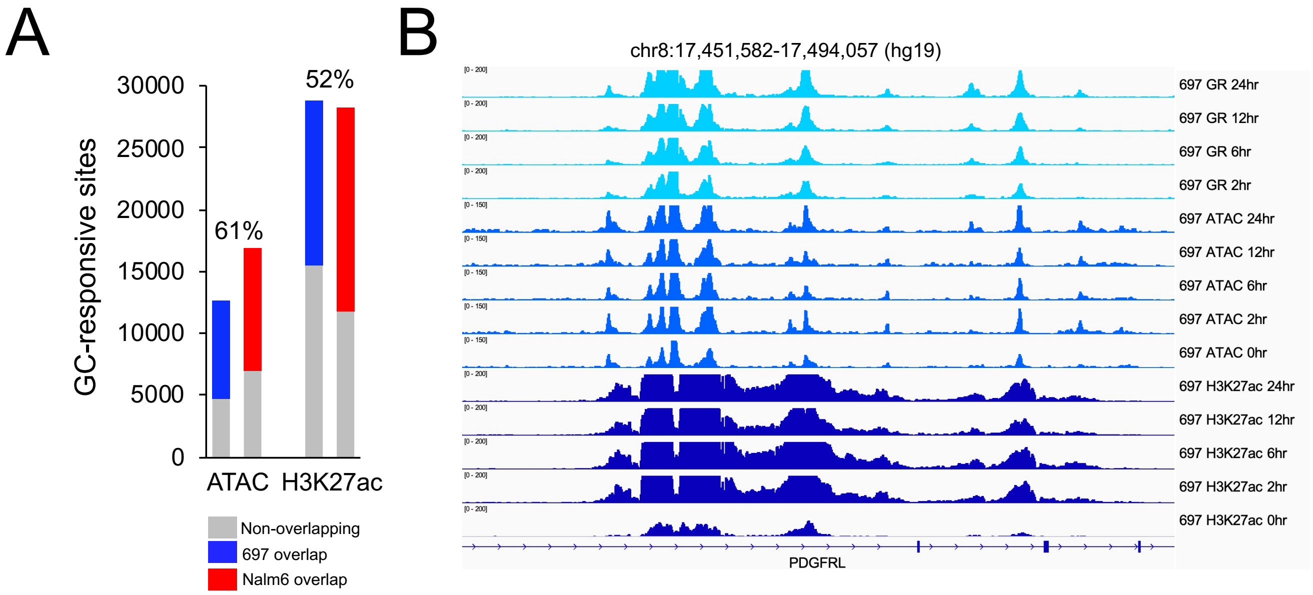

Supplementary Figure 6. (A) Bar graph depicts the number of GC-responsive ATAC-seq accessible chromatin sites (ATAC) and GC-responsive H3K27ac sites in 697 cells (first) and Nalm6 cells (second). The number of GC-responsive sites that overlap with sites of GR occupancy is shown in 697 cells (blue) and Nalm6 (red) cells and the fraction of total GC-responsive ATAC-seq and H3K27ac sites with GR occupancy is provided above. **(B)** IGV browser track of 697 GR, ATAC-seq and H3K27ac across the time course at the *PDGFRL* gene locus. Sites of GR occupancy are present at GC-responsive accessible chromatin sites that exhibit enhanced accessibility following GC treatment and/or GC-responsive H3K27ac sites that exhibit enhanced enrichment following GC treatment.

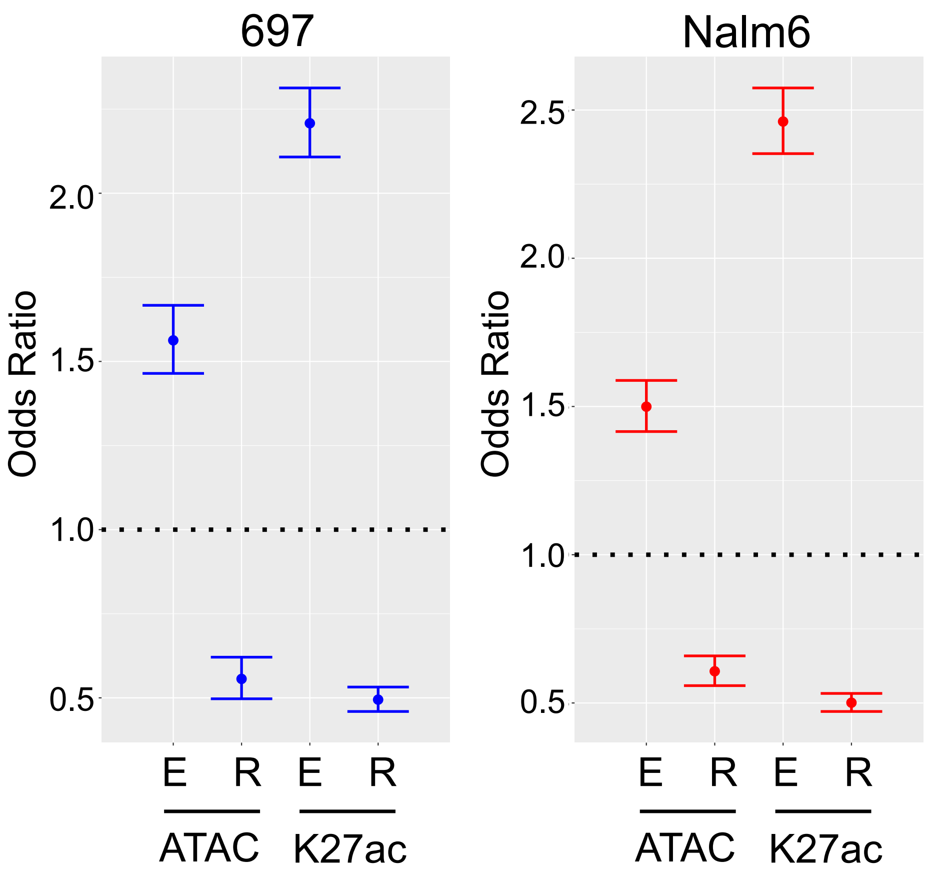

Supplementary Figure 7. Odds ratio showing the enrichment of HGRs with GREs for different chromatin hallmarks in 697 (left) and Nalm6 (right) cells. E=enhanced, R=reduced, ATAC=ATAC-seq, K27ac=H3K27ac.

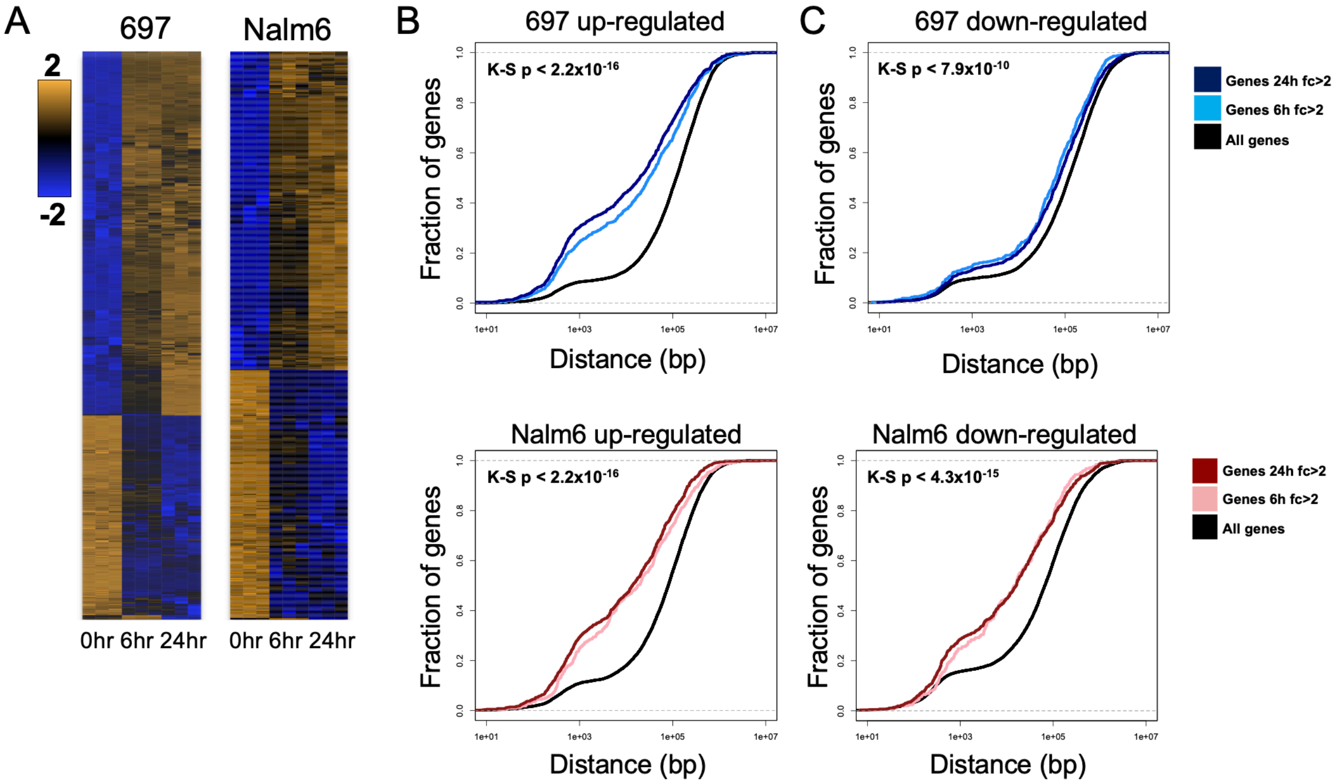

Supplementary Figure 8. (A) Heatmap depicts all differentially expressed (fold change >2) genes after prednisolone treatment. A majority of genes show linear increases or decreases in expression during the time course. **(B)** Cumulative distribution functions of GREs exhibiting enhanced H3K27ac enrichment near up-regulated genes at 6hr and 24hr timepoints. All expressed genes are shown in black and used as a background for comparisons. Kolmogorov-Smirnov (K-S) test p-values are provided. **(C)** Cumulative distribution functions of HGRs exhibiting reduced H3K27ac enrichment near down-regulated genes at 6hr and 24hr timepoints. All expressed genes are shown in black and used as a background for comparisons. Kolmogorov-Smirnov (K-S) test p-values are provided.

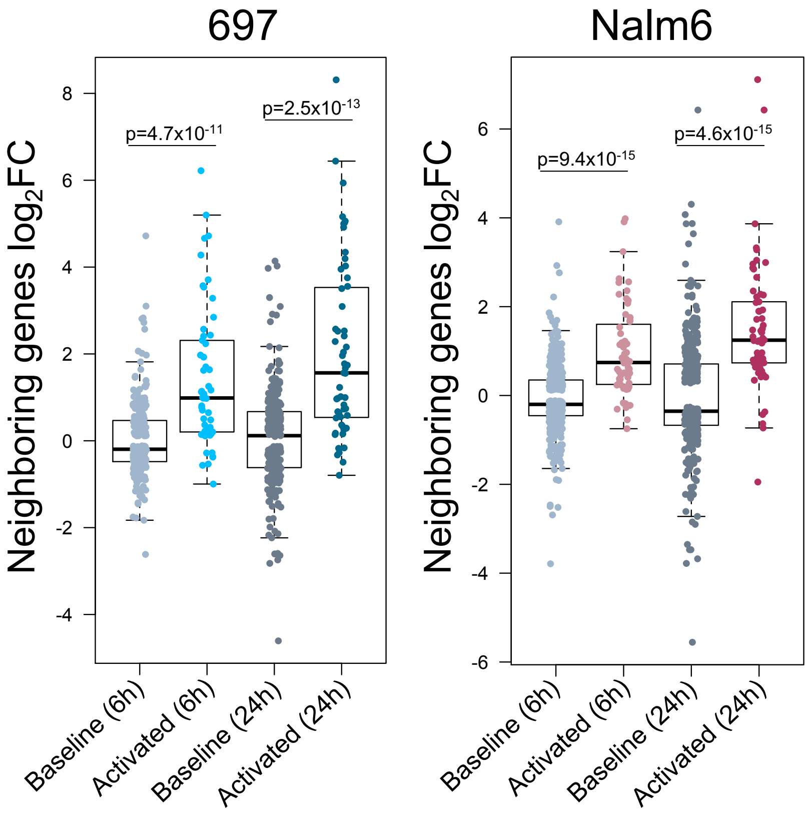

Supplementary Figure 9. Log_2 fold change following GC treatment of genes associated with SEs that are pre-established (Baseline) and genes associated with GC-responsive SEs formed after GC treatment (Activated) in 697 (left) and Nalm6 (right) cells. Log_2 fold changes of DEGs (FDR<0.01) at 6hr and 24hr following GC treatment is provided. Wilcoxon p-values show significant up-regulation of genes associated with GC-responsive SEs compared to pre-established SEs at each timepoint and in each cell.

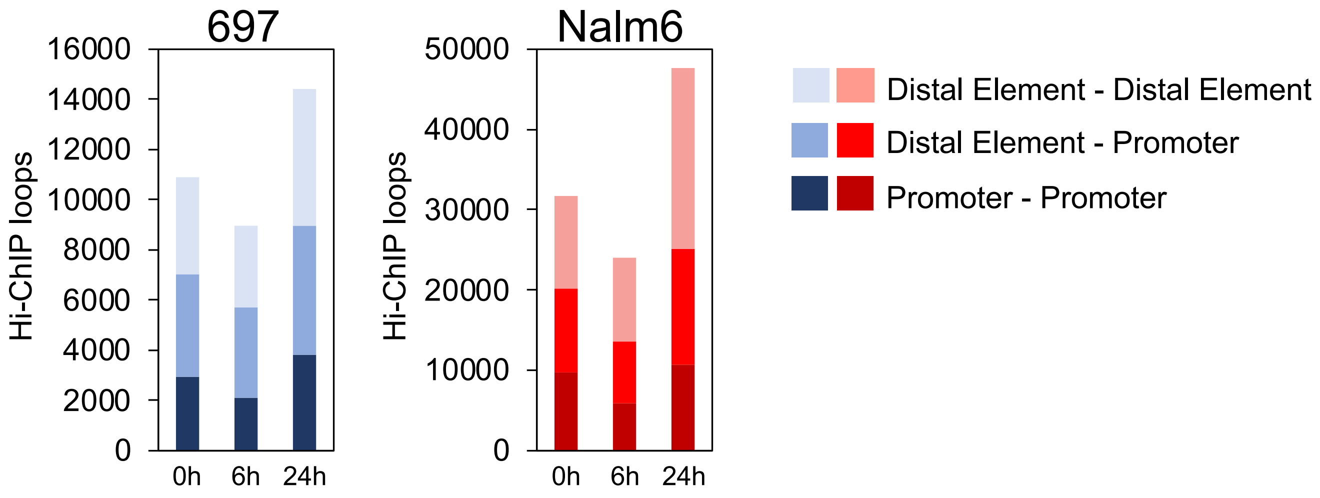

Supplementary Figure 10. H3K27ac HiChIP loops in 697 and Nalm6 cells. The number of HiChIP loops between distal elements, distal elements and promoters and between promoters is provided.

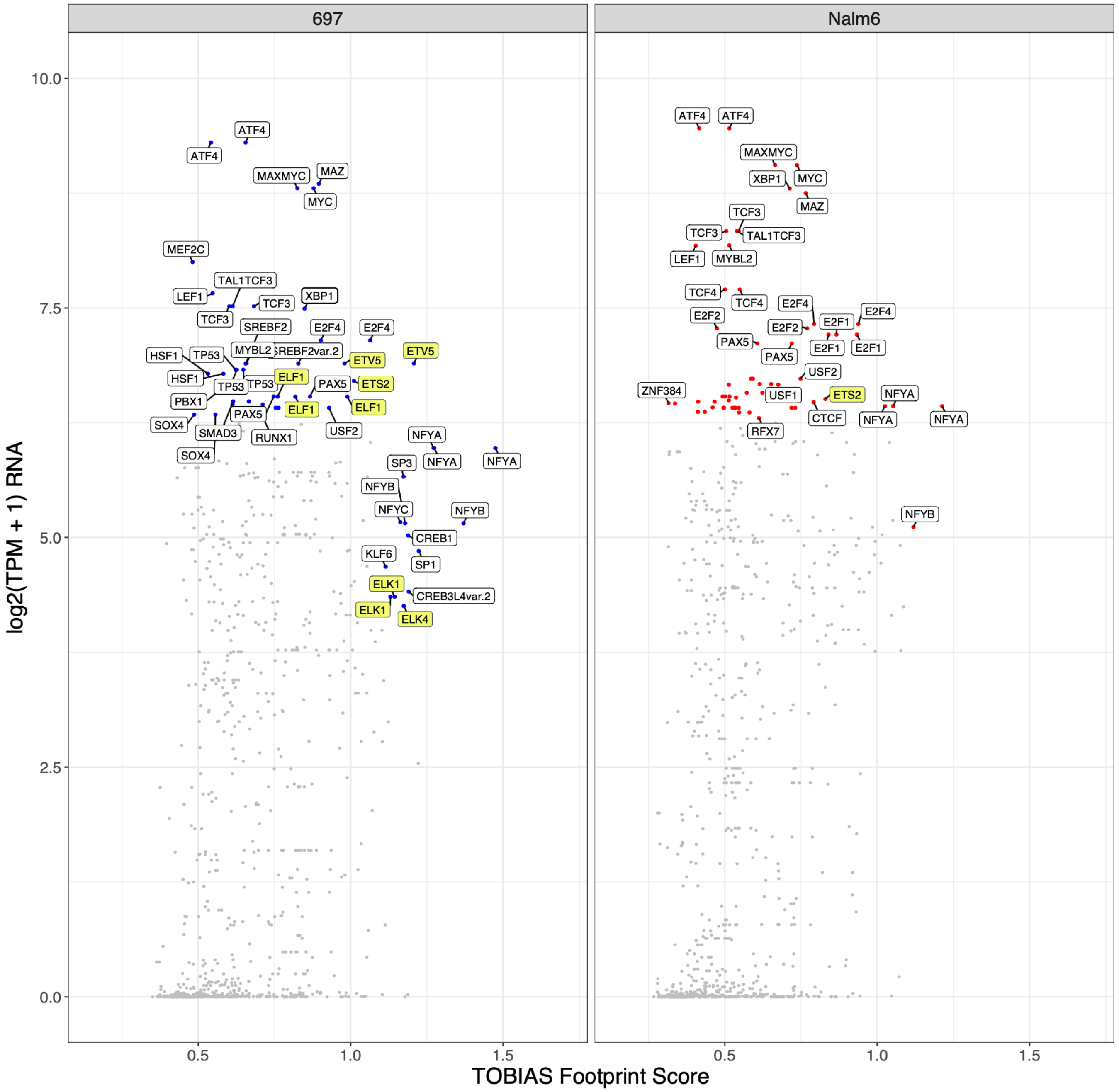

Supplementary Figure 11. Candidate cooperating TFs of GR are shown for 697 (left) and Nalm6 (right) cells. TOBIAS footprint scores (x-axis) were compared to gene expression (y-axis) obtained from 0hr RNA-seq data.

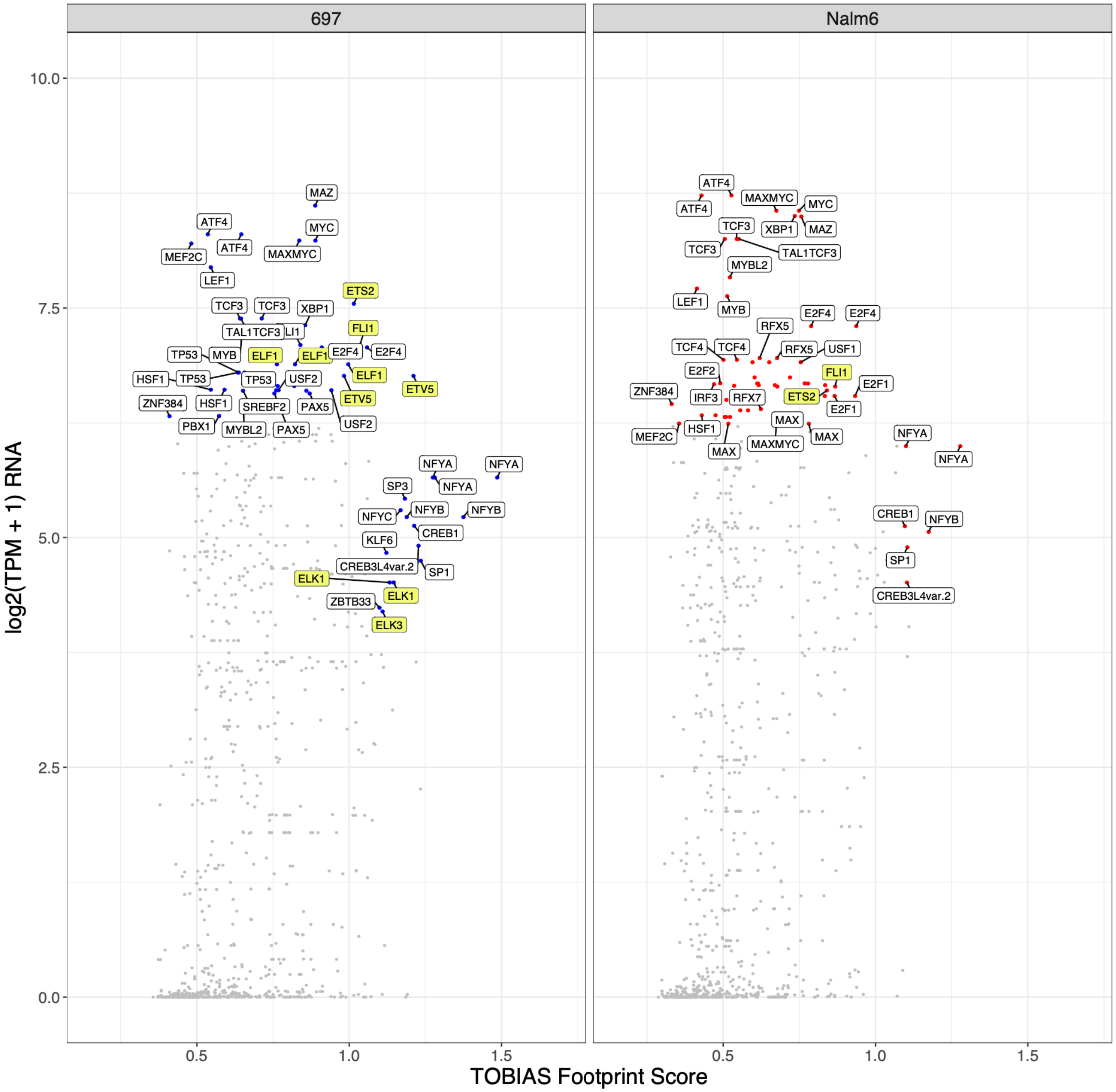

Supplementary Figure 12. Candidate cooperating TFs of GR are shown for 697 (left) and Nalm6 (right) cells. TOBIAS footprint scores (x-axis) were compared to gene expression (y-axis) obtained from 6hr RNA-seq data.

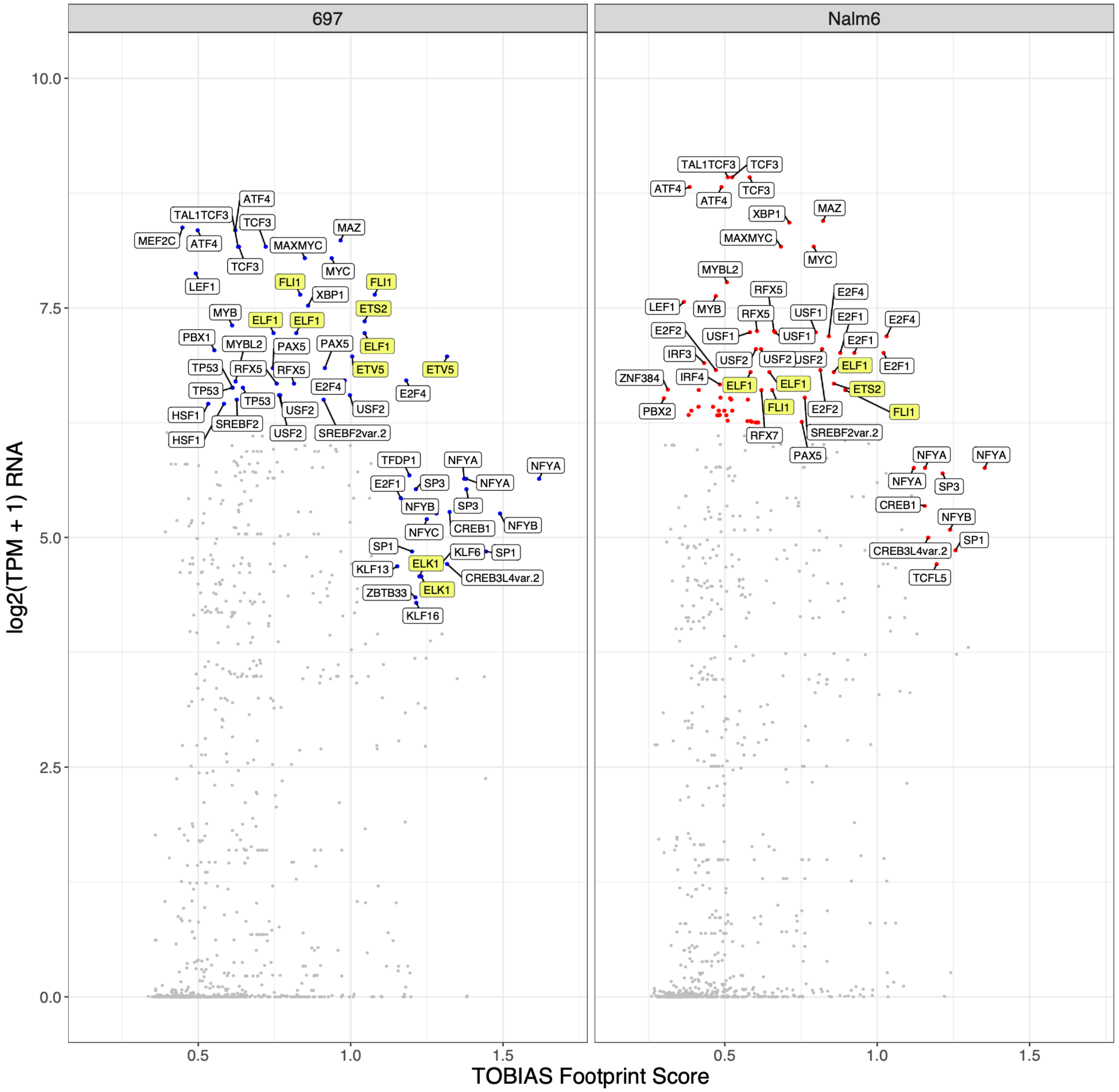

Supplementary Figure 13. Candidate cooperating TFs of GR are shown for 697 (left) and Nalm6 (right) cells. TOBIAS footprint scores (x-axis) were compared to gene expression (y-axis) obtained from 24hr RNA-seq data.

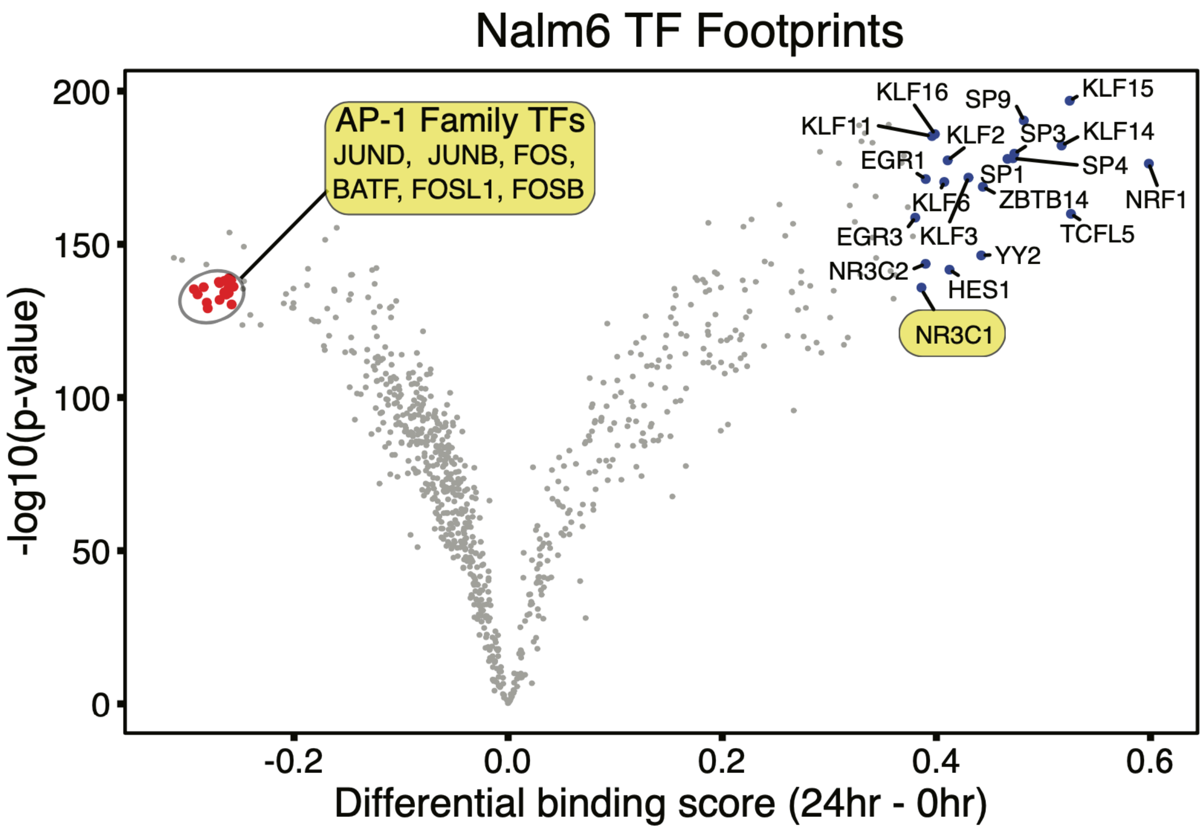

Supplementary Figure 14. TOBIAS TF footprint score differences (x-axis) and their significance (y-axis) between 0hr and 24hr timepoints in Nalm6 is shown cells. Significant AP-1 family TF footprints with stronger scores at 0hr are highlighted and denoted in red. Several outlier TFs with significantly stronger scores at 24hr are denoted in blue, including GR or NR3C1 which is also highlighted.

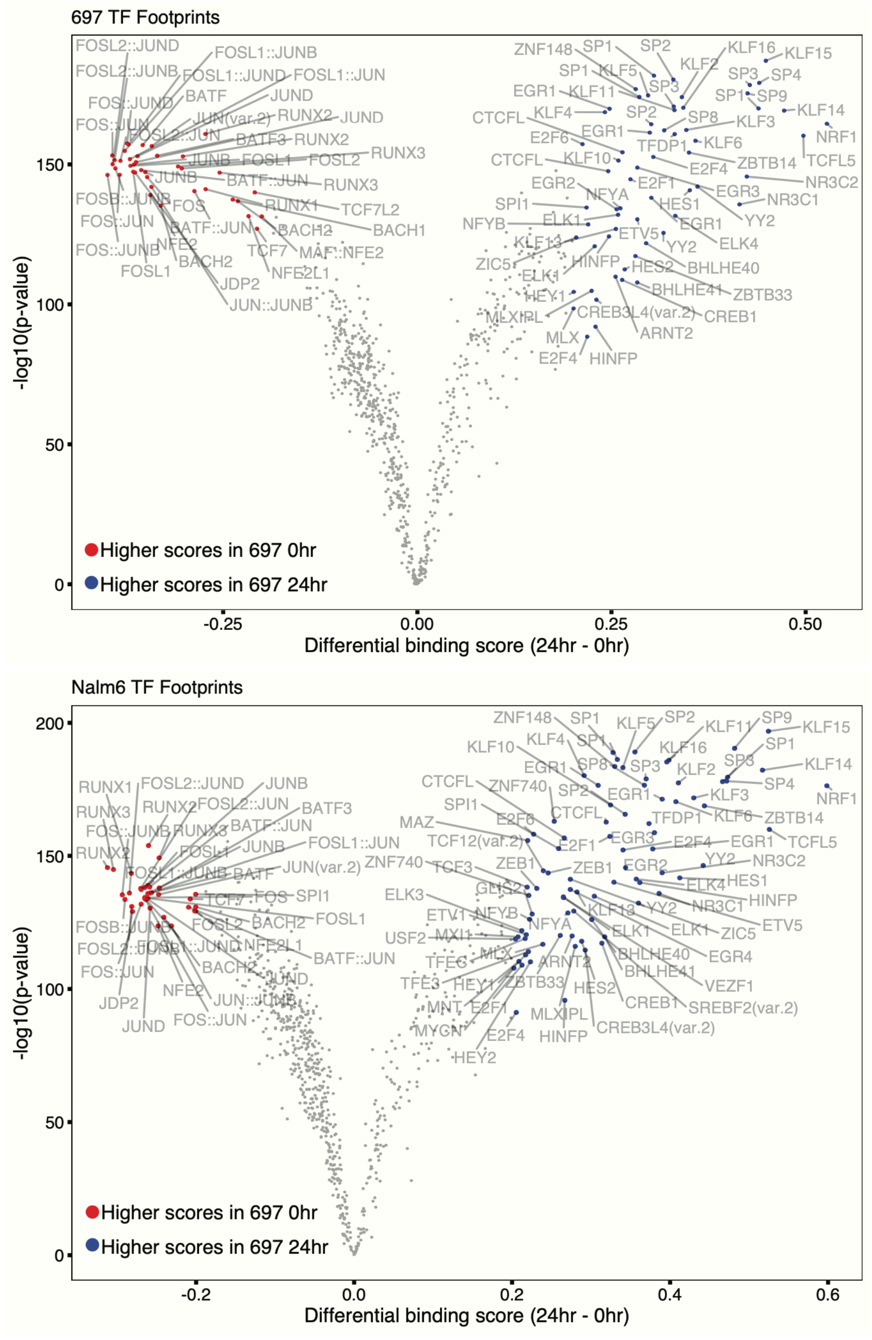

Supplementary Figure 15. TOBIAS TF footprint score differences (x-axis) and their significance (y-axis) between 0hr and 24hr timepoints in 697 (top panel) and Nalm6 (bottom panel) cells. Red denotes significant TF footprints with stronger scores at 0hr and blue denotes significant TF footprints with stronger scores at 24hrs.

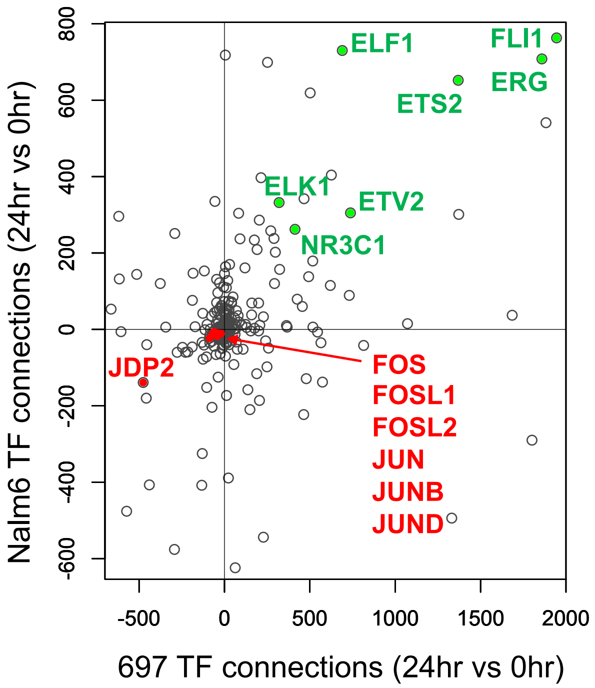

Supplementary Figure 16. Comparison between 697 (x-axis) and Nalm6 (y-axis) cells of changes in the number of PECA TF-connected genes (gene connectivity) at 24hrs versus 0hrs for individual TFs. Notable TFs are highlighted. GR is represented by NR3C1.

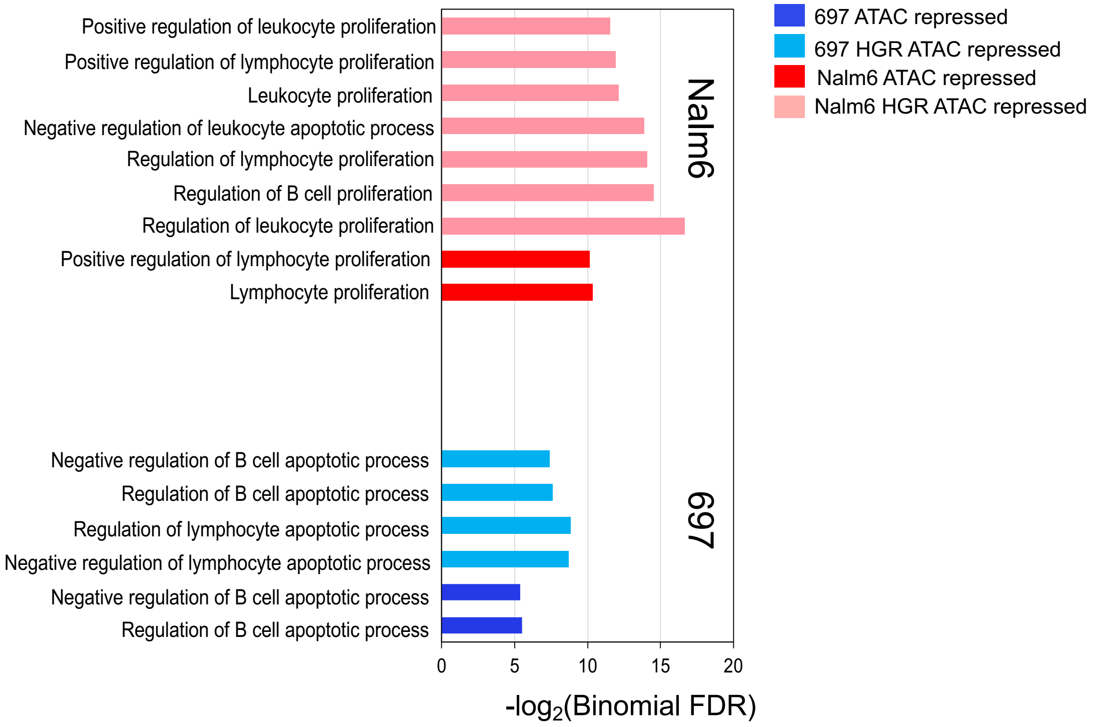

Supplementary Figure 17. Gene Ontology pathway enrichment analysis of AP-1 footprints at GC-responsive reduced accessible chromatin sites (ATAC repressed) and GC-responsive reduced accessible chromatin HGRs (HGR ATAC repressed) in 697 and Nalm6 cells.

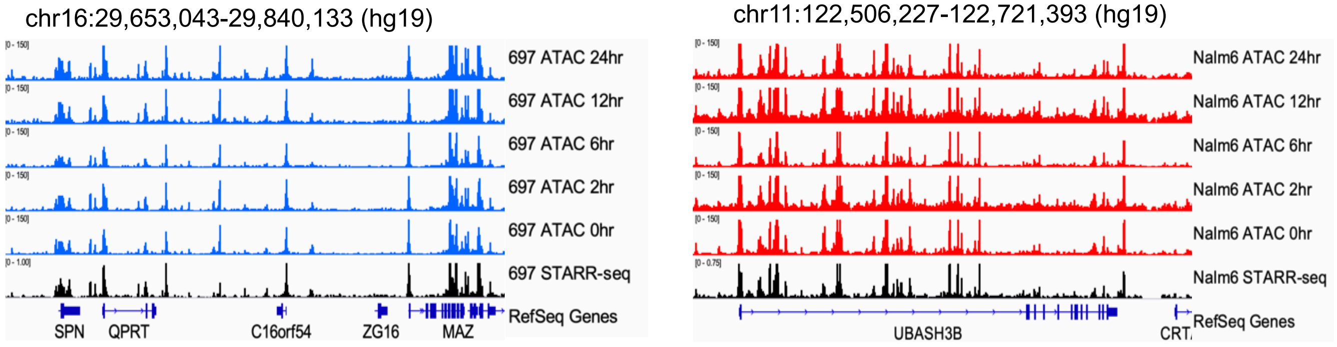

Supplementary Figure 18. 697 (left) and Nalm6 (right) IGV browser tracks of ATAC-seq during the time course and ATAC-STARR-seq plasmid DNA input is provided. ATAC-seq tracks denote read counts and ATAC-STARR-seq tracks are shown as read counts per million.

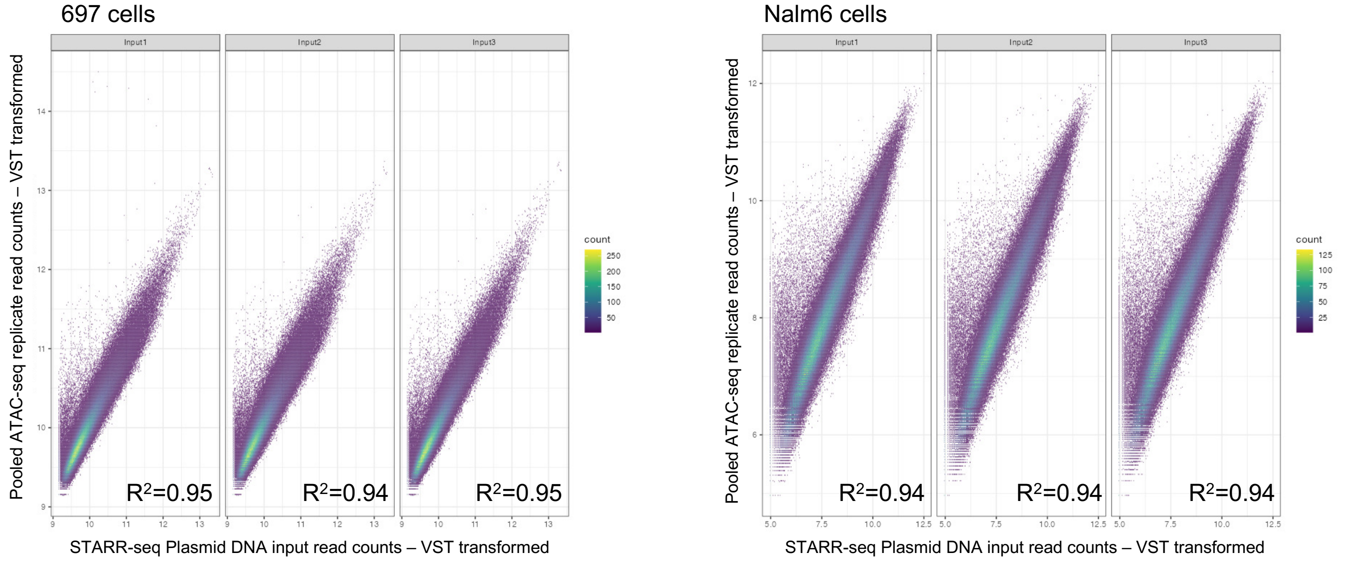

Supplementary Figure 19. Plots depict the correlation of normalized read counts between ATAC-STARR-seq plasmid input DNA (x-axis) and 3 pooled ATAC-seq replicates (y-axis) in 697 (left) and Nalm6 (right) cells.

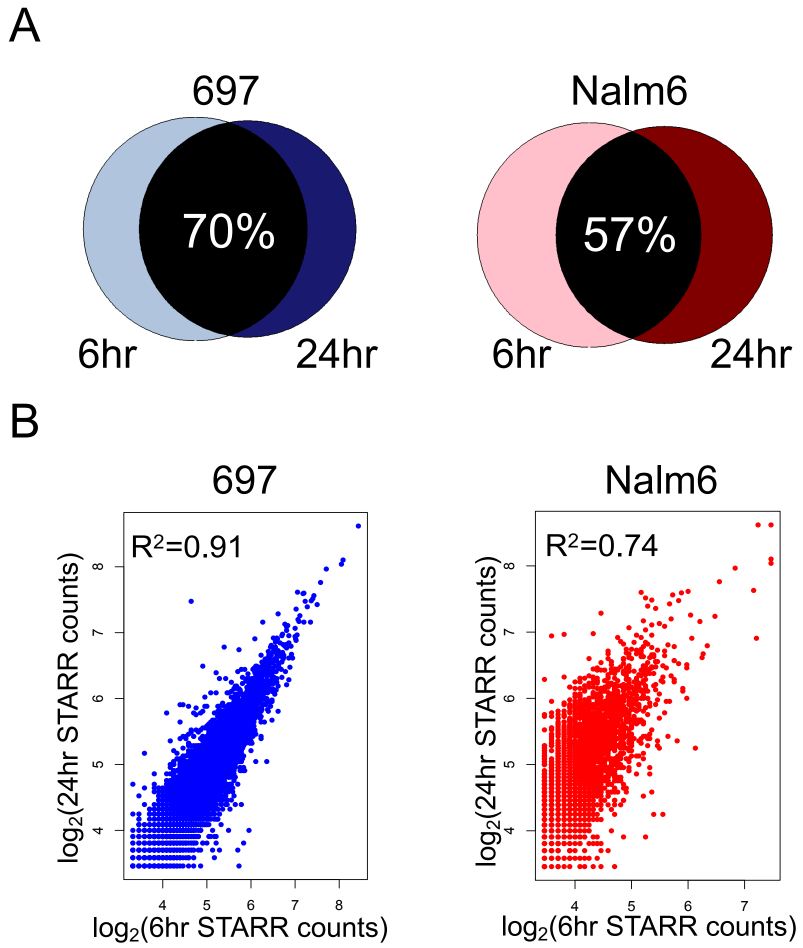

Supplementary Figure 20. (A) Venn diagrams display the overlap between active STARR-seq sites at 6hr and 24hr in 697 (top) and Nalm6 (bottom) cells. **(B)** Read count correlation of active STARR-seq sites identified after 6hr (x-axis) and after 24hr (y-axis) of prednisolone treatment in 697 (left) and Nalm6 (right) cells.

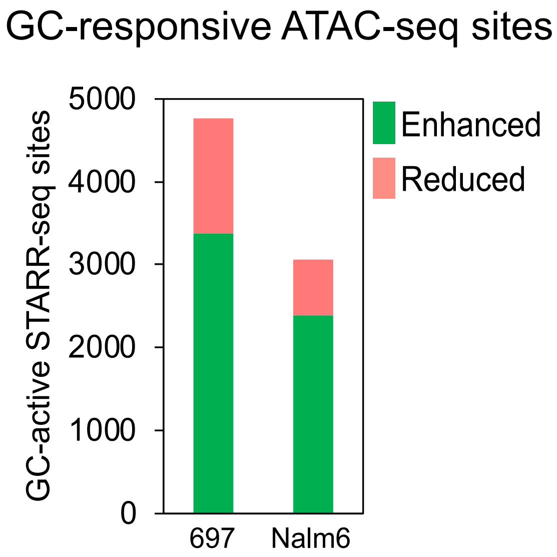

Supplementary Figure 21. Total number of GC-active STARR-seq sites that are GC-responsive ATAC-seq sites exhibiting enhanced (green) or reduced (pink) open chromatin accessibility following prednisolone treatment is shown.

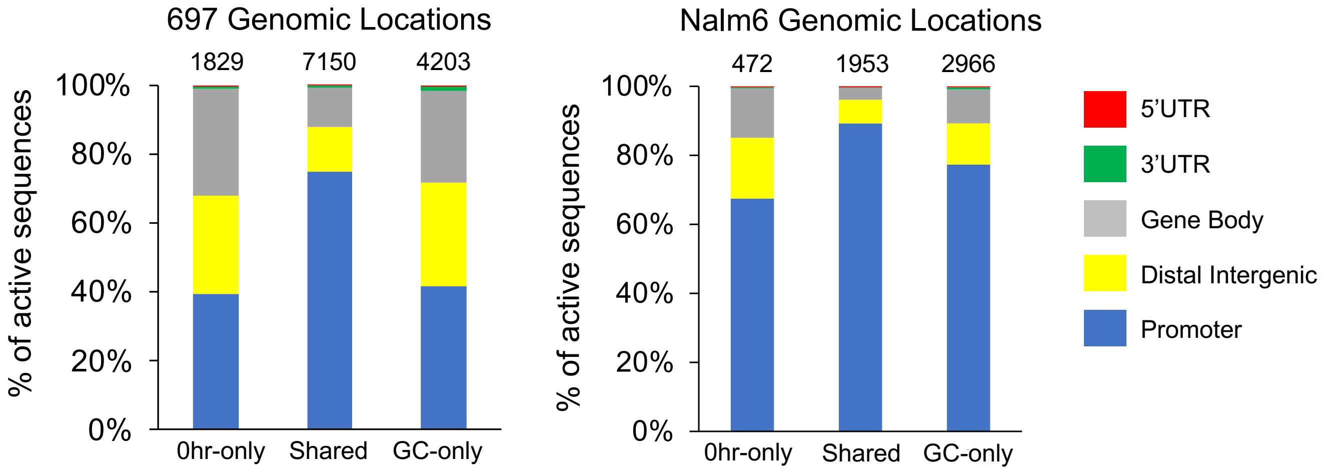

Supplementary Figure 22. Genomic locations of 697 (left) and Nalm6 (right) active STARR-seq sites that are shared (Shared) between 0hr and GC (6+24hr) timepoints and that are specific to 0hr (0hr-only) or GC (GC-only) timepoints is provided. The total number of sites for each category are provided.

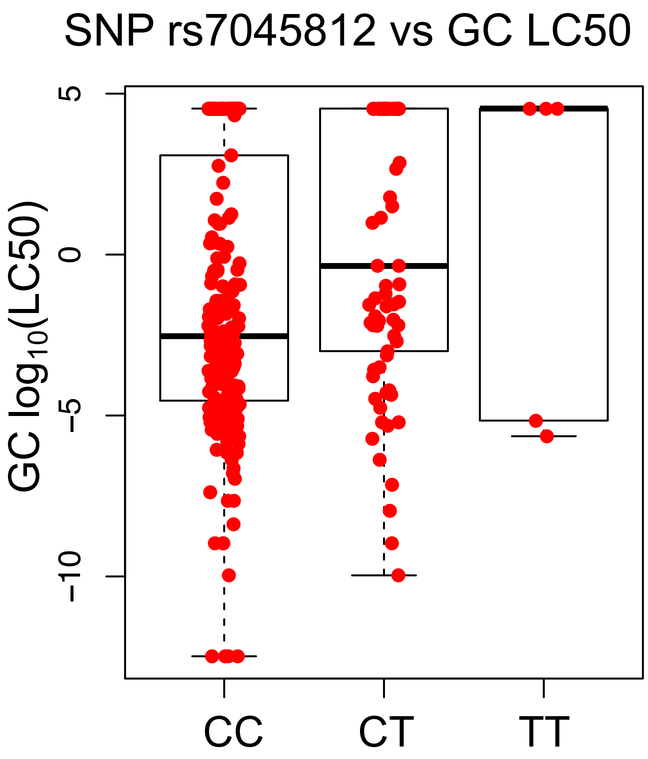

Supplementary Figure 23. Plot showing the effects of rs7045812 alleles on LC50 GC sensitivity in primary ALL cells from the Total XVI St. Jude patient cohort. Individuals heterozygous or homozygous for the alternative T allele exhibit stronger GC resistance compared to homozygotes harboring the reference C allele.

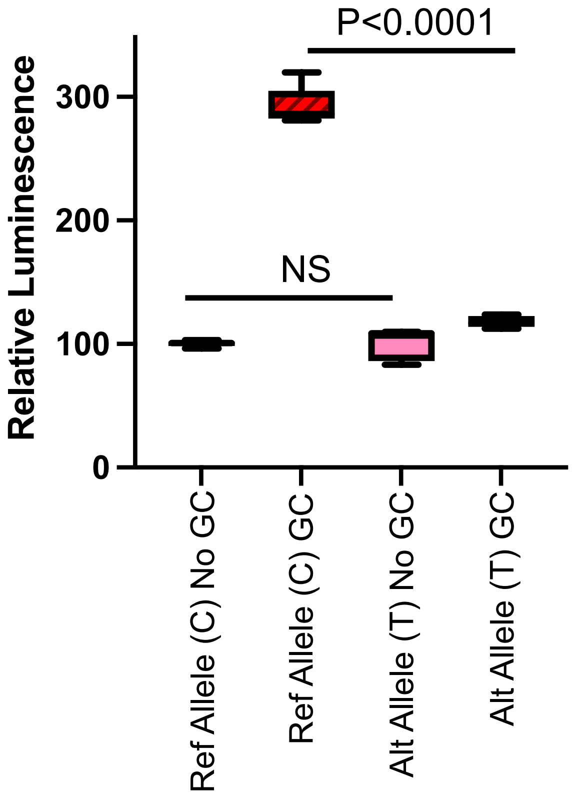

Supplementary Figure 24. Luciferase reporter assay testing a 300-bp fragment of DNA in the opposite orientation centered on the reference C allele and alternative T allele of rs7045812 in the presence or absence of prednisolone (5uM; n=5 per group).

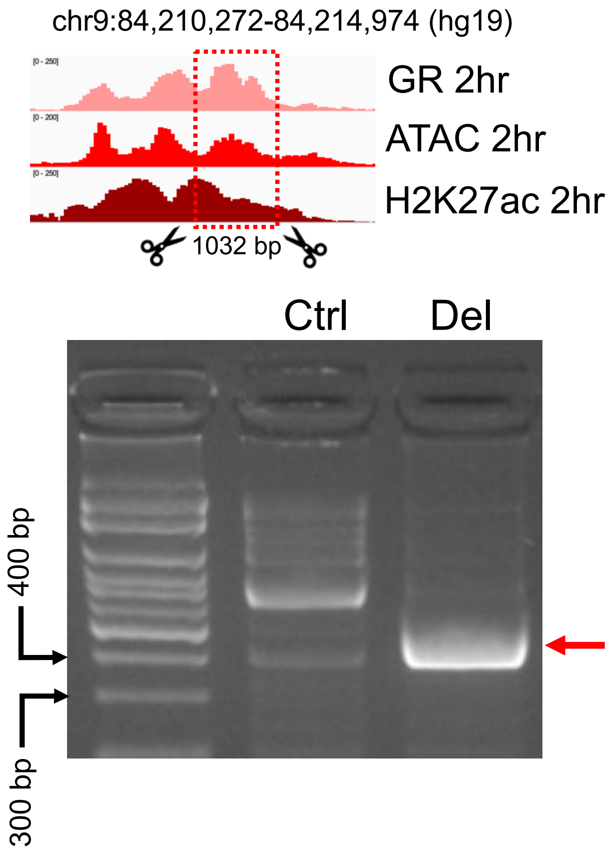

Supplementary Figure 25. CRISPR/Cas9 deletion of the *TLE1* HGR spanning rs7045812. The location of Cas9 nuclease cut sites is shown above and PCR validation of deletion (Del) using primers flanking cut sites is shown below (red arrow).

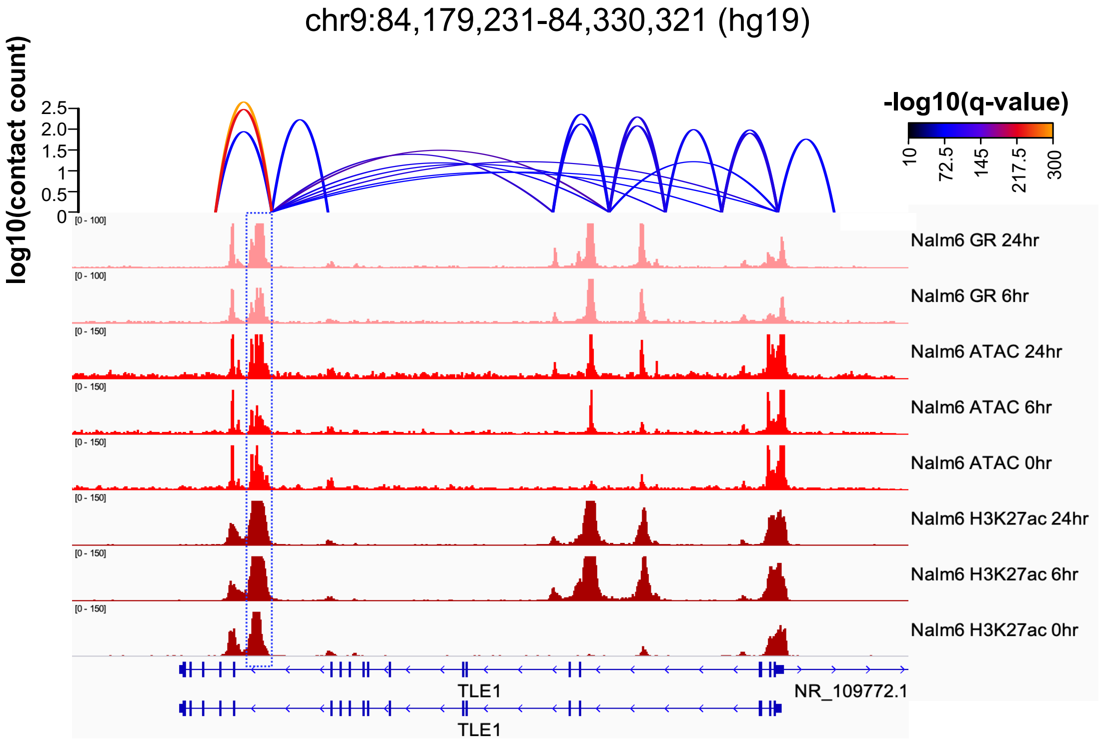

Supplementary Figure 26. IGV browser image of H3K27ac HiChIP loops between the *TLE1* HGR spanning rs7045812 and the *TLE1* promoter.

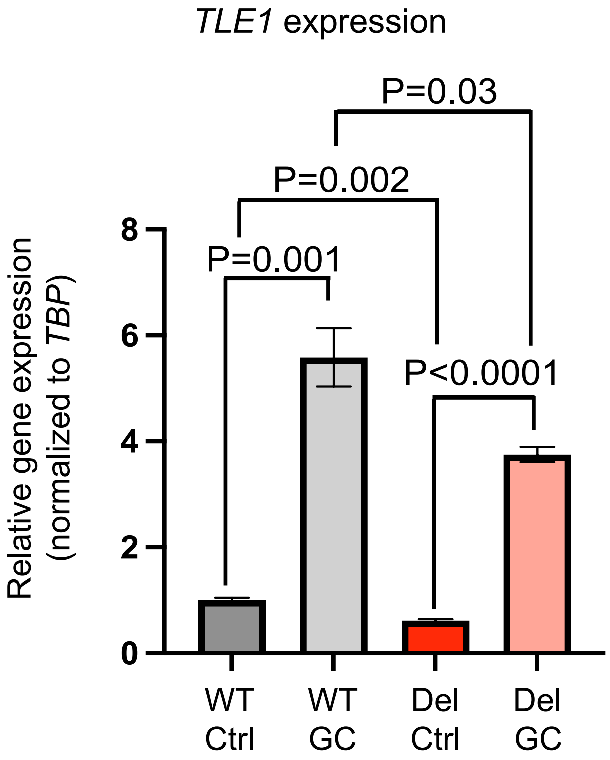

Supplementary Figure 27. *TLE1* RT-qPCR results of parental/wild-type (WT) and *TLE1* HGR deleted (Del) Nalm6 cells is shown in the presence (GC) and absence (Ctrl) of prednisolone (5uM; n=6 per group).

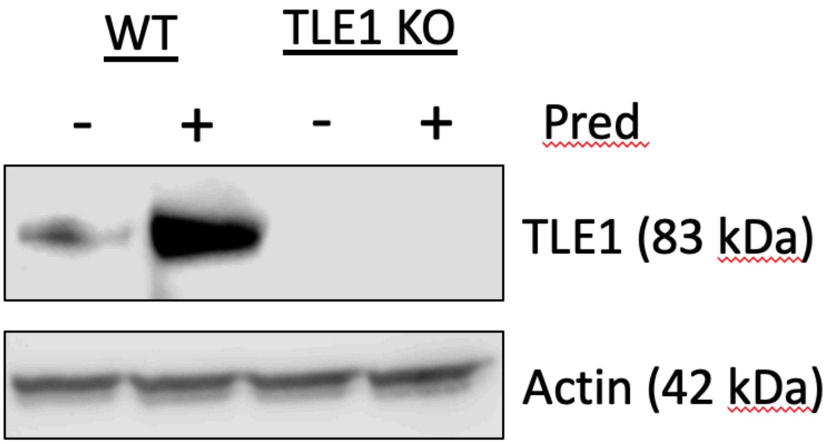

Supplementary Figure 28. Western blot shows TLE1 protein expression from *TLE1* knockout Nalm6 cells (KO) and wild-type Nalm6 cells (WT) following prednisolone treatment (5uM).

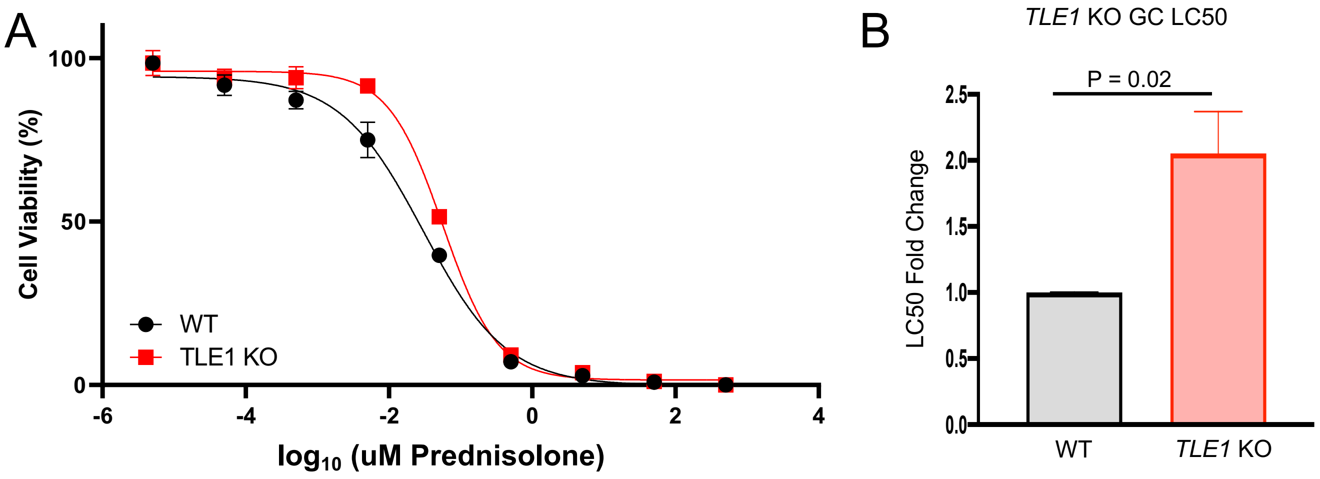

Supplementary Figure 29. (A) Prednisolone drug response curves in *TLE1* knockout Nalm6 cells (KO) and wild-type Nalm6 cells (WT). Cell viability is provided on the Y-axis and $\log_{10}$ prednisolone concentration is shown on the X-axis. Concentrations of prednisolone used were: 5pM, 50pM, 0.5nM, 5nM, 50nM, 0.5uM, 5uM, 50uM and 500uM. **(B)** LC50 fold change is shown in *TLE1* gene knockout (KO) Nalm6 cells compared to parental/wild-type (WT) Nalm6 cells after 72-hours of prednisolone treatment (n=3 per group).

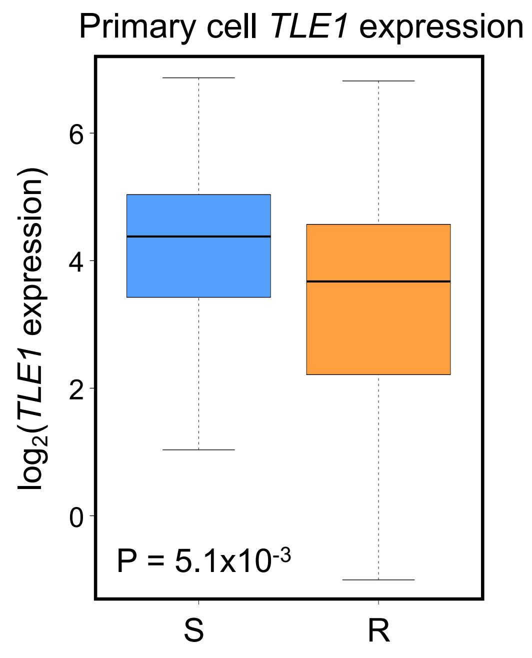

Supplementary Figure 30. Expression of *TLE1* in GC sensitive (n=142) and GC resistant (n=93) primary ALL cells from patients.

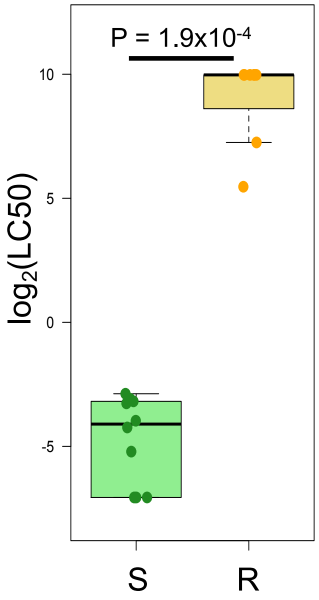

Supplementary Figure 31. Log_2 of prednisolone LC50 between GC-sensitive (n=10) and GC-resistant (n=9) primary ALL cells from patients. P-value is provided for differences in LC50 between GC-sensitive and GC-resistant samples.

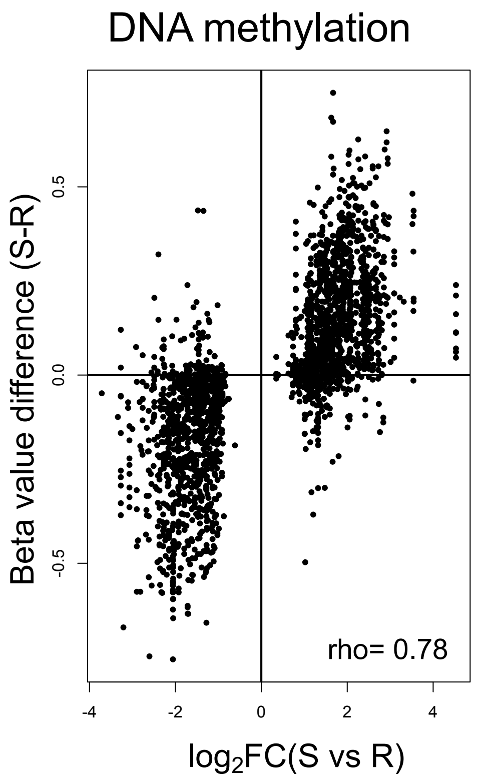

Supplementary Figure 32. CpG DNA methylation beta values differences between GC-sensitive and GC-resistant primary ALL cells compared to $\log_2$ fold change in chromatin accessibility between GC-sensitive and GC-resistant primary ALL cells at all overlapping GC-resistance open chromatin sites are plotted (S=sensitive, R=resistant).

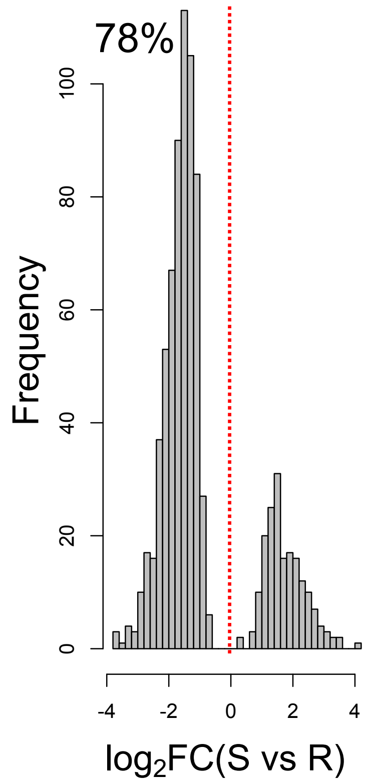

Supplementary Figure 33. Histogram showing changes in chromatin accessibility between GC-resistant and GC-sensitive primary ALL samples from patients at GC-resistance open chromatin sites that map to HGRs (S=sensitive, R=resistant).

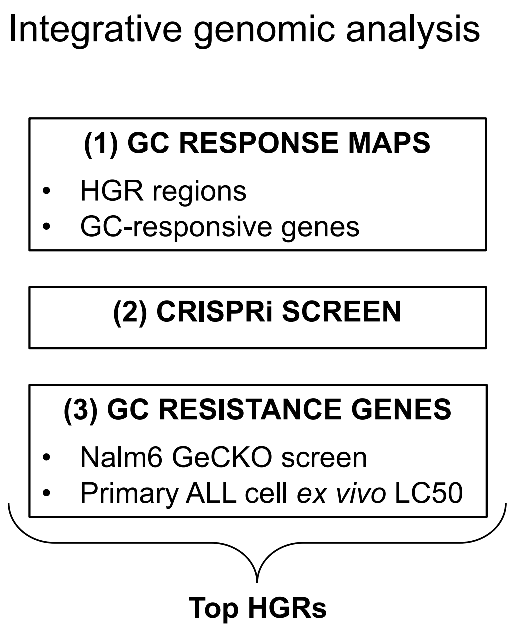

Supplementary Figure 34. Overview of the integrative genomic analysis and associated datasets used to identify top HGRs.

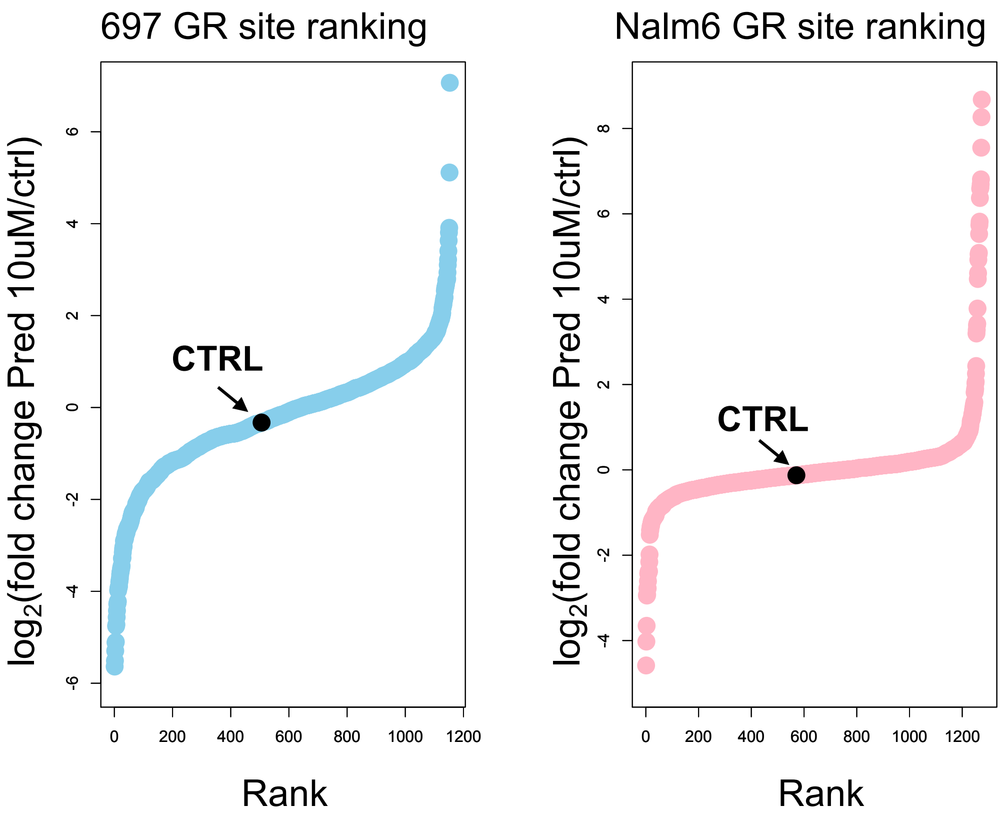

Supplementary Figure 35. Ranking of CRISPRi log_2 fold enrichment through aggregation of sgRNA results at each site of GR occupancy site in 697 (left) and Nalm6 (right) cells by is provided. The aggregate control sgRNA rank is labeled in black. All GR occupancy sites with log_2 fold enrichment >0 were further utilized in integrative analyses.

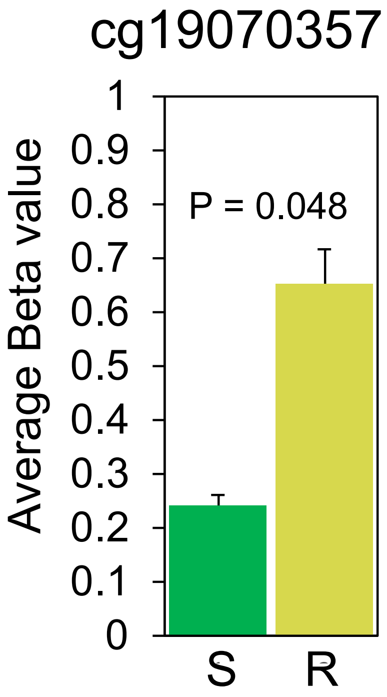

Supplementary Figure 36. Average DNA methylation beta values between GC-sensitive and GC-resistant patient samples at a CpG site that maps to HGR Peak1585.

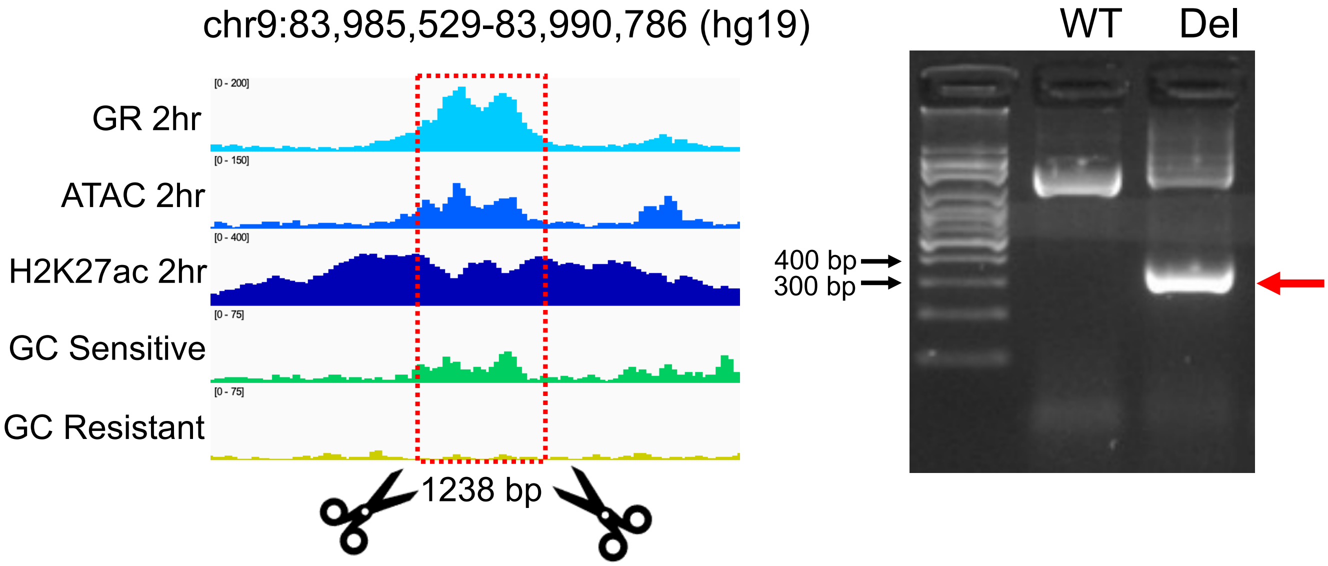

Supplementary Figure 37. The location of Cas9 nuclease cut sites is shown on the left and PCR validation of deletion using primers flanking cut sites is shown on the right for *TLE1* Peak1585 deletion (red arrow).

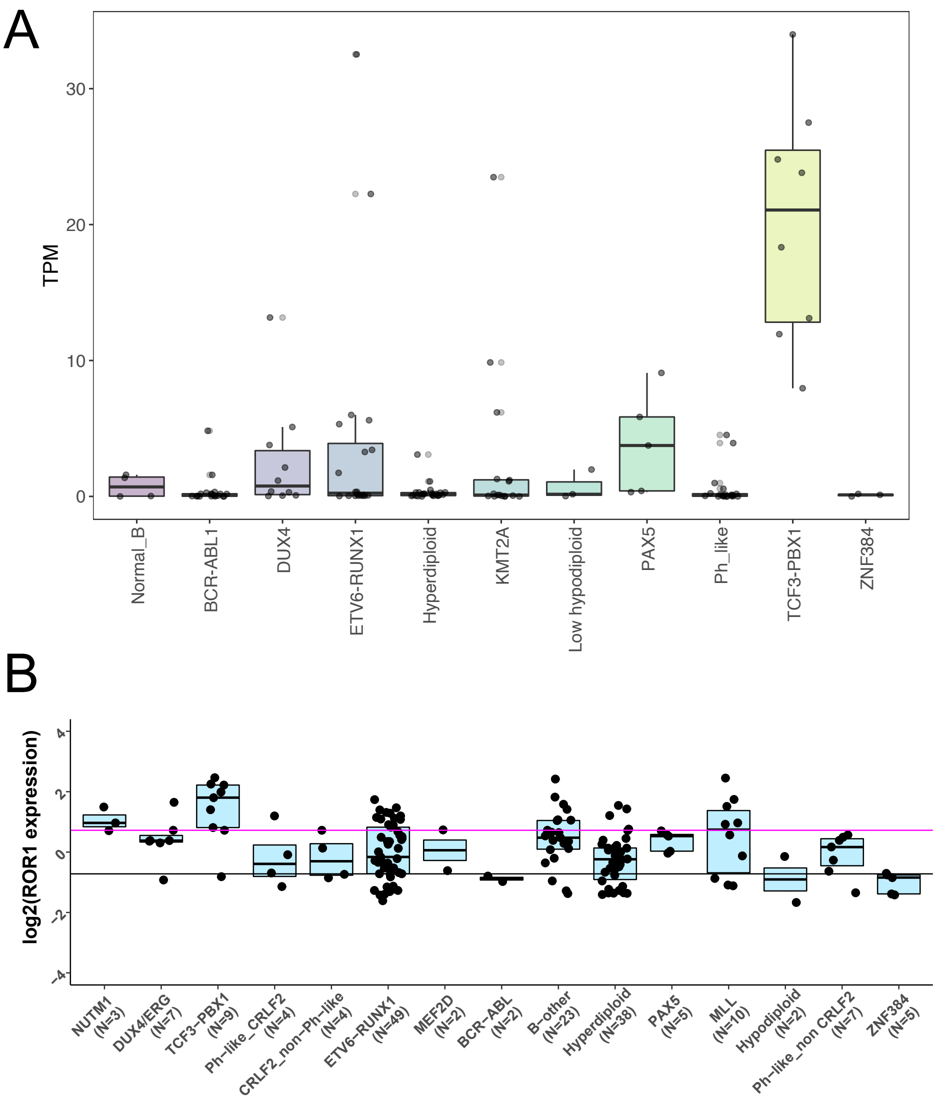

Supplementary Figure 38. *ROR1* expression is shown for two different patient cohorts using RNA-seq transcripts per million or TPM **(A)** and microarray data **(B)**. The expression of *ROR1* in normal B-cells is shown in **(A)** as well.

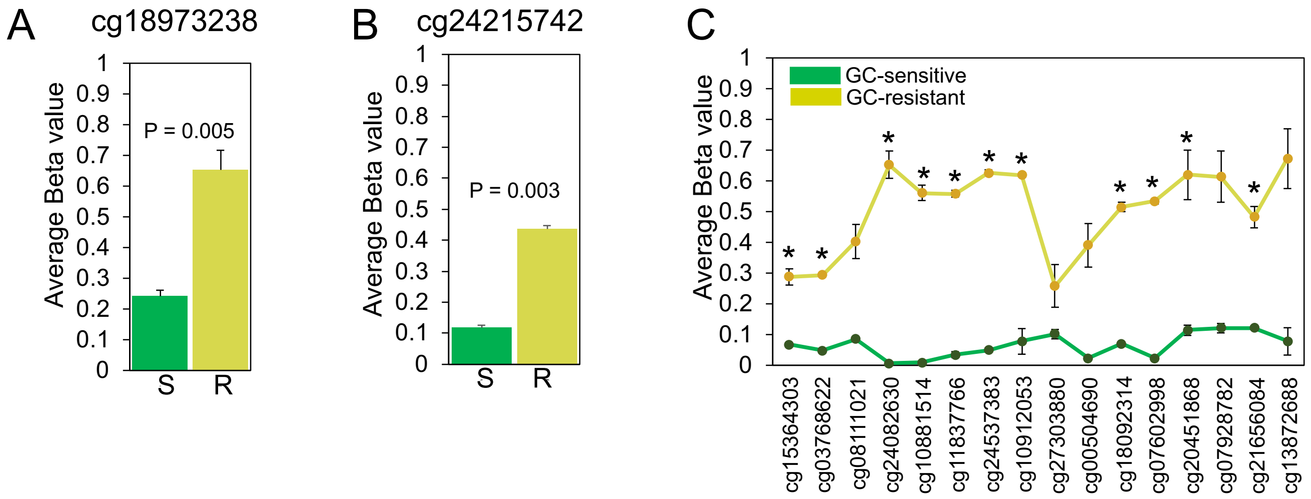

Supplementary Figure 39. Average DNA methylation beta values between GC-sensitive and GC-resistant patient samples for a CpG site at *ROR1* Peak42 **(A)**, a CpG site at *ROR1* Peak43 **(B)** and multiple CpG sites at *ROR1* promoter Peak44 **(C)**. *p<0.05.

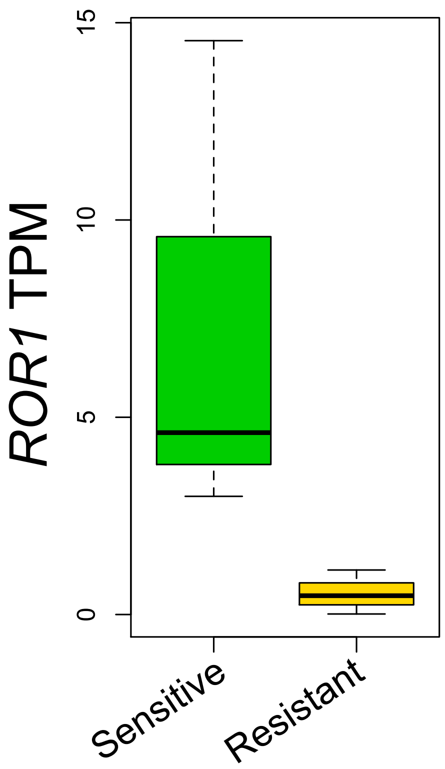

Supplementary Figure 40. *ROR1* transcripts per million (TPM) in GC-sensitive (ETV6-RUNX1 subtype, n=3) and GC-resistant (ETV6-RUNX1 subtype, n=3) primary ALL samples with ATAC-seq data.

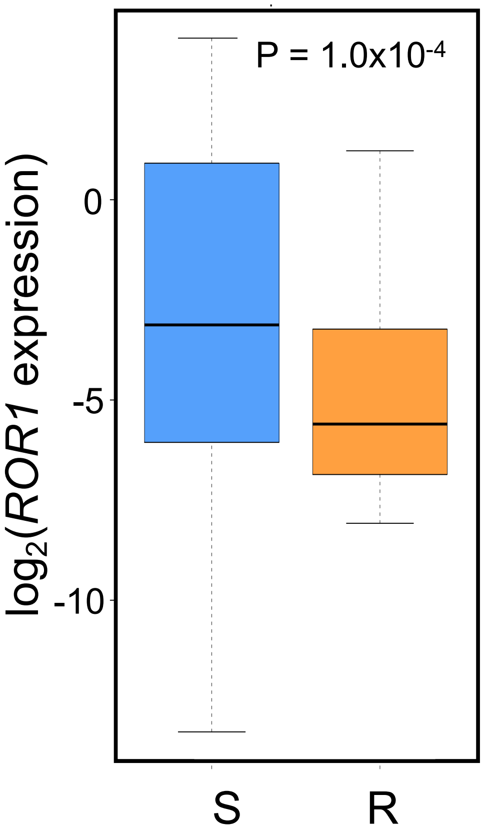

Supplementary Figure 41. *ROR1* gene expression in GC-sensitive (n=142) and GC-resistant (n=93) primary ALL cells from patients. *ROR1* expression is higher in GC-sensitive patients compared to GC-resistant patients.

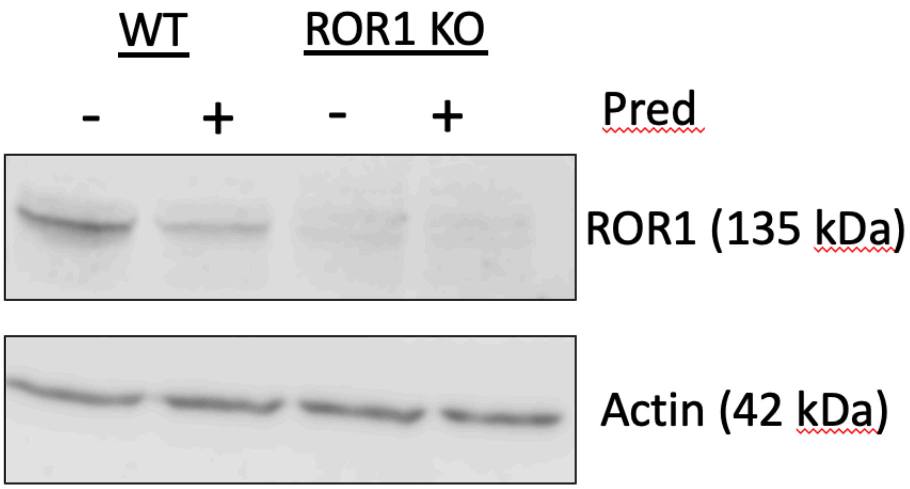

Supplementary Figure 42. Western blot shows ROR1 protein expression from partial *ROR1* knockout in 697 cells (KO) and wild-type 697 cells (WT) following prednisolone treatment (10uM).

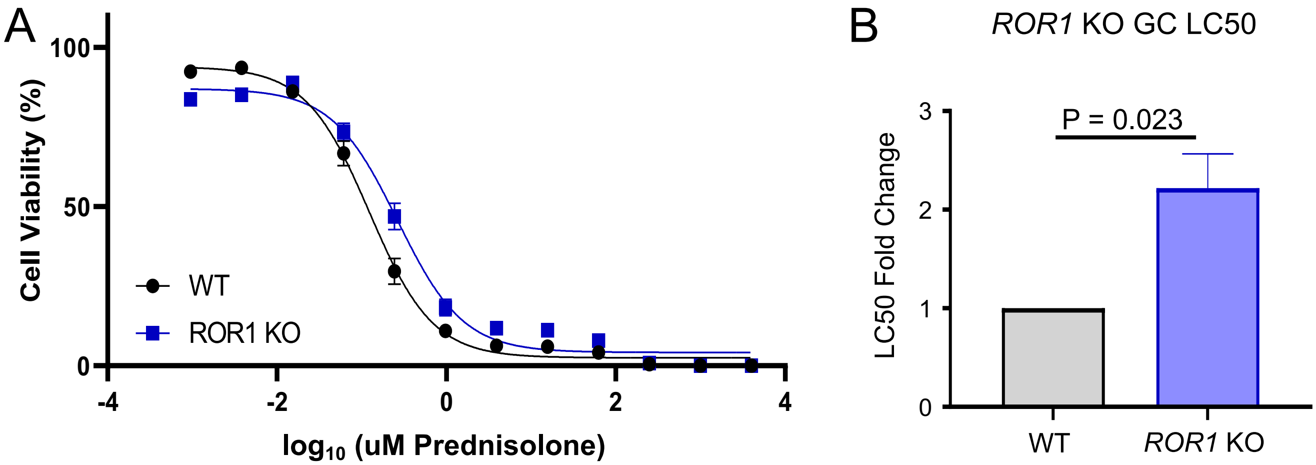

Supplementary Figure 43. (A) Prednisolone drug response curves in partial *ROR1* knockout 697 cells (KO) and wild-type 697 cells (WT). Cell viability is provided on the Y-axis and $\log_{10}$ prednisolone concentration is shown on the X-axis (n=4 per group). Concentrations of prednisolone used were: 0.95nM, 3.8nM, 15.2nM, 61nM, 244nM, 976nM, 3.9uM, 15.6uM, 62.5uM, 250uM, 1000uM, 4000uM. **(B)** LC50 fold change is shown in partial *ROR1* knockout (KO) 697 cells compared to parental/wild-type (WT) 697 cells after 72-hours of prednisolone treatment (n=4 per group).

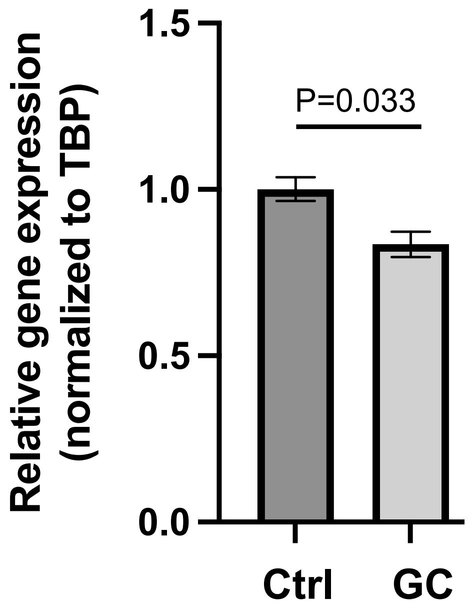

Supplementary Figure 44. RT-qPCR results of *ROR1* relative gene expression in wild-type/parental 697 cells treated with vehicle control (Ctrl) or prednisolone (GC, 10uM) (n=6 per group).

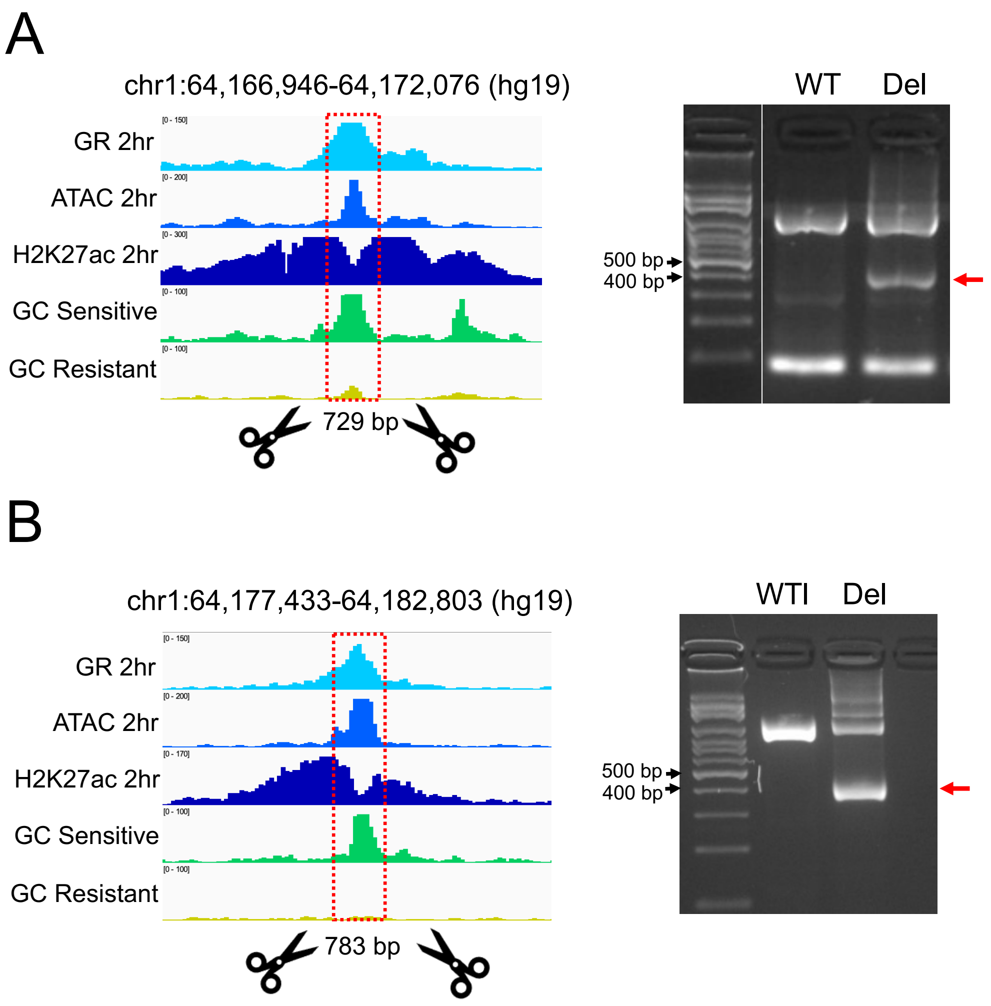

Supplementary Figure 45. The location of Cas9 nuclease cut sites is shown above and PCR validation of deletion using primers flanking cut sites is provided below for *ROR1* Peak42 **(A)** and *ROR1* Peak43 **(B)** deletions (red arrows).

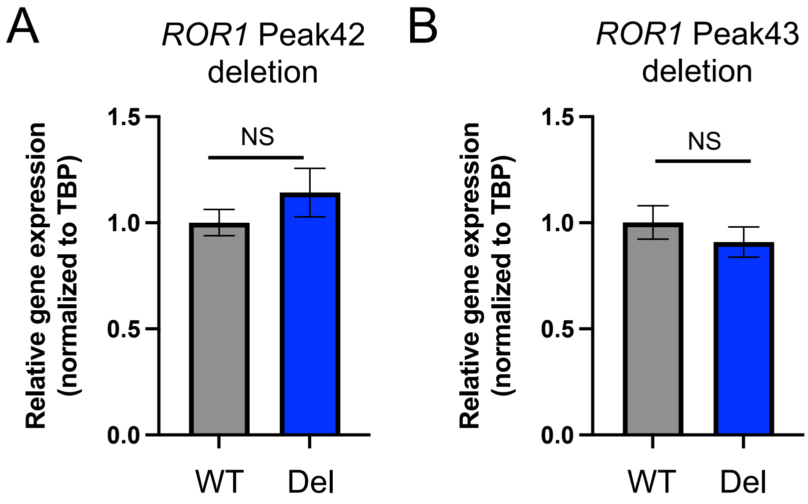

Supplementary Figure 46. RT-qPCR results of baseline *ROR1* gene expression in parental/wild-type (WT) and *ROR1* Peak42 **(A)** and *ROR1* Peak43 **(B)** deleted (Del) 697 cells in the absence of prednisolone (n=6 per group).
