# Supplementary material for: Epigenomic profiling of glucocorticoid responses identifies cis-regulatory disruptions impacting steroid resistance in childhood acute lymphoblastic leukemia
Source: Leukemia. Author manuscript; Available in PMC 2022 Oct 4. (PMC9522591; doi:10.1038/s41375-022-01685-z)
Supplement: 1833642_Sup_Methods [file NIHMS1833642-supplement-1833642_Sup_Methods.docx]

**SUPPLEMENTARY METHODS**

**Epigenomic profiling of glucocorticoid responses identifies *cis*-regulatory disruptions impacting steroid resistance in childhood acute lymphoblastic leukemia**

Brennan P. Bergeron^1,2,3,§^, Jonathan D. Diedrich^1,2,§^, Yang Zhang^4^, Kelly R. Barnett^1,2^, Qian Dong^1,2^, Daniel C. Ferguson^1,2^, Robert J. Autry^1,2,5^, Wenjian Yang^1,2^, Baranda S. Hansen^6^, Colton Smith^1,2^, Kristine R. Crews^1,2^, Yiping Fan^7^, Ching-Hon Pui^1,5,8^, Shondra M. Pruett-Miller^3,6^, Mary V. Relling^1,2,3^, Jun J. Yang^1,2,3,5^, Chunliang Li^3,4^, William E. Evans^1,2,3^ and Daniel Savic^1,2,3,5,*^

^1^ Hematological Malignancies Program and Center for Precision Medicine in Leukemia, St. Jude Children’s Research Hospital, Memphis, TN 38105, USA.

^2^Department of Pharmacy and Pharmaceutical Sciences, St. Jude Children’s Research Hospital, Memphis, TN 38105, USA.

^3^Graduate School of Biomedical Sciences, St. Jude Children’s Research Hospital, Memphis, TN 38105, USA.

^4^Department of Tumor Cell Biology, St. Jude Children’s Research Hospital, Memphis, TN 38105, USA.

^5^Integrated Biomedical Sciences Program, University of Tennessee Health Science Center, Memphis, TN 38105, USA.

^6^Department of Cell and Molecular Biology and Center for Advanced Genome Engineering, St. Jude Children’s

Research Hospital, Memphis, TN 38105, USA.

^7^Department of Computational Biology, St. Jude Children’s Research Hospital, Memphis, TN 38105, USA.

^8^Department of Oncology, St. Jude Children’s Research Hospital, Memphis, TN 38105, USA.

^§^ Equal contributions

^*^Corresponding author

**Corresponding Author:**

Daniel Savic, Ph.D.

Department of Pharmacy and Pharmaceutical Sciences

Division of Pharmaceutical Sciences

St. Jude Children’s Research Hospital

262 Danny Thomas Place, MS: 313

Phone: 901-595-5346

Email: [daniel.savic@stjude.org](mailto:daniel.savic@stjude.org)

**Human ALL cell lines**

697 and Nalm6 ALL cell lines were purchased from Deutsche Sammlung von Mikroororganisem und Zellkulturen (DKMZ). Both cell lines have been authenticated by STR profiling and tested for mycoplasma contamination.

**H3K27ac HiChIP**

Chromatin was fixed with disuccinimidyl glutarate (DSG) and formaldehyde. Fixed chromatin was digested *in situ* with micrococcal nuclease (MNase) then extracted upon cell lysis. Chromatin fragments were incubated with H3K27ac antibody overnight for chromatin immunoprecipitation. The antibody-protein-DNA complex was pulled down with protein A/G-coated beads. Chromatin ends were repaired and ligated to a biotinylated bridge adapter followed by proximity ligation of adapter containing ends. After proximity ligation, crosslinks were reversed, and the DNA was purified from proteins and converted into a sequencing library. The sequencing library was generated using Illumina-compatible adapters. Biotin-containing fragments were isolated using streptavidin beads before PCR enrichment of the library. The library was sequenced on an Illumina HiSeqX platform.

Sequences were aligned to a referenced genome (hg19) with bwa mem (v0.7.17) with the -5SP settings enabled. The resulting alignments were pipped into pairtools (v0.3.0) to be parsed with a mapQ filter of 5 imposed, --walks-policy was set to 5 unique, and a –max-inter-align-gap set to 30, then sorted and deduped. The alignment and pairs QC statistics were compiled by pairtools. The filtered, deduplicated and sorted alignment was then converted into both .pairs file and .sam file with the split function. The resulting sam was converted to bam and sorted with samtools (v1.7) view and sort functions. The final bam file was indexed with samtools. The .pairs file was used as input for loop calling with FitHiChIP (v9.0)^1^ along with the required modules (samtools v1.9, bowtie2 v2.3.5.1, macs2 v2.1.1, htslib v1.9, hic-pro v2.11.4, bedtools v2.29.2, R v4.1.0) using the recommended parameters for H3K27ac datasets and the bin size set to 10kb.

**ATAC-STARR-seq**

FAST-ATAC was performed in 697 and Nalm6 cells treated with prednisolone (697= 10uM and Nalm6= 5uM) or vehicle control. Four replicates of 10,000 cells were collected at 0-,2-,6-,12-, and 24-hour time points and subsequent PCR was performed using custom HPLC-purified primers (F: 5′-TAGAGCATGCACCGGCAAGCAGAAGACGGCATACGAGATNNNNATGTCTCGTGGGCTCGGAGATGT-3′, R**:** 5′-GGCCGAATTCGTCGATCGTCGGCAGCGTCAGATGTG-3′, where NNNN corresponds to a random 4 nt i7 barcode sequence) and NEBNext Ultra II Q5 Master Mix (NEB #M0544S). Thermocycler conditions were as follows: 98°C for 30 s, then 5 cycles of: 98°C for 10 s and 65°C for 75 s. To reduce GC and size bias in PCR, the appropriate number of PCR cycles is determined using qPCR following the same thermocycler conditions, but with 20 cycles instead of 5. Amplified transposed DNA fragments were double size selected using SPRI Select beads (Beckman Coulter #B23317) using a 1-0.65 bead ratio. An aliquot of each sample was submitted for BioAnalyzer QC to verify correct fragment size selection. All 20 samples were pooled together and concentrated using a SpeedVac for subsequent cloning reactions.

The hSTARR-seq_ORI vector used to generate the plasmid library for each cell line was a gift from Alexander Stark (Addgene plasmid #99296). The linear backbone was generated by splitting 10ug of hSTARR-seq_ORI across 10 separate reactions and digesting the vector overnight with AgeI-HF and SalI-HF restriction enzymes (NEB #R3552S and R3138S). This was followed by gel excision (Qiagen #28704) to extract a linear 2.5kb fragment corresponding to the hSTARR-seq_ORI backbone. Cloning of the fragment library was completed using the NEBuilder HiFi DNA Assembly Cloning Kit (NEB E5520S) with 25 individual HiFi DNA Assembly cloning reactions utilizing a 3:1 molar ratio of insert to vector backbone. Each group of five HiFi DNA Assembly reactions was collected and cleaned up using the Qiagen MinElute Enzymatic Reaction cleanup kit (Qiagen #28204), eluted in 10 μL of dH2O, and transformed into five 20 μL aliquots of MegaX DH10B T1R Electrocomp Cells (Invitrogen #C640003) following the manufacturer’s protocol. The 22 μL of bacteria/DNA mix was placed in a 1mm cuvette (MidSci #EC1) and electroporated with a Gene Pulser Xcell Total Electroporation System (BioRad #1652660) using the conditions: 2.0kV, 200 Ω, 25μF with electroporation time constants ranging between 4.92 and 5.08ms. Bacteria were allowed to recover in 1 mL of pre-heated (37°C) Recovery Medium for 1hr while shaking at 225rpm at 37°C. After recovery, samples were pooled and diluted in 2L of Luria broth and 100μg/mL of ampicillin to be grown overnight (10-11 hr while shaking at 225rpm and 37°C). Overnight cultures were split into 500 mL aliquots to maxiprep using the PureLink HiPure Plasmid Filter Maxiprep Kit (Invitrogen #K210017), eluted into 350 μL of dH2O, and pooled together (1.4mL total of eluted library per cell line). Multiple 10ng aliquots of library were amplified with HPLC-purified primers (F: 5′-CAAGCAGAAGACGGCATACGAGAT-3′, R: 5′-AATGATACGGCGACCACCGAGATCTACAC[X8]TCGTCGGCAGCGTC-3′; “X8” sequence corresponds to sample barcode, chosen from Illumina Nextera barcode list) using the aforementioned PCR conditions, cleaned up using a Qiagen MinElute PCR purification kit (Qiagen #28004), and submitted for sequencing to ensure library complexity. Upon verification of library complexity, eluted plasmid DNA libraries were concentrated using the SpeedVac to a 5ug/ul concentration for downstream transfections.

697 and Nalm6 cells were grown in RPMI 1640 Medium (Gibco #21870-076) and supplemented with 10% FBS Certified One Shot (Gibco #A31604-01), 1% glutamine (Gibco #25030-081), and 1% PenStrep (Gibco #15070-063) while maintaining a density between 1x10^6^ and 2x10^6^ cells/mL and cellular viability above 90% with culture media changes every 3 days. Prior to transfections, cell were passaged a minimum of 3 times in culture medium containing 10% Charcoal:Dextran Stripped FBS (GeminiBio #100-119) in place of the aforementioned FBS Certified One Shot. Cells were split prior to transfections to ensure 90% confluency and a minimum of 95% viability on the day of electroporation. Electroporations were completed using a MaxCyte STX Instrument alongside the CL-1.1 Processing Assembly following the manufacturer’s standard recommendations. CL-1.1 Processing Assemblies were used to electroporate 3.5x10^8^ cells that had been pre-washed with room temperature PBS and resuspended with plasmid DNA (100μg/mL) in 3.5 mL of EP Buffer. Cells were electroporated using the MaxCyte STX Optimization 2 Protocol and immediately transferred to a T175 to incubate for 20 minutes at 37°C with 5% CO2. After incubation, 120 mL of the aforementioned RPMI + stripped FBS medium was added to the T175 and cells were allowed to recover overnight. PKR and TBK1/IKK inhibitors were added directly after resuspension at a final concentration of 1μM per inhibitor to prevent induction of interferon-stimulated genes that has been observed in similar STARR-seq assays^2^. A total of 4 bulk transfections using 3.5x10^8^ cells were performed for each cell line. The following morning each bulk transfection of cells were split into 3 replicates and treated with prednisolone (697=10uM and Nalm6=5uM; 6hr or 24hr) or vehicle control and collected. Collected cells were washed with PBS and resuspended in RLT/BME for total RNA isolation (Qiagen #75144) with the optional DNase treatment (Qiagen #79254). From total RNA, mRNA was isolated using the Oligotex mRNA Midi Kit (Qiagen #70042). A second DNase treatment with Turbo DNase (Thermo Fisher #AM1907) was completed before cleaning up and concentrating the mRNA (Qiagen #74204). cDNA was generated with Superscript III Reverse Transcriptase (Thermo Fisher #18080085) using a gene-specific RT primer (5′-CAAACTCATCAATGTATCTTATCATG-3′) that is located in the sgGFP reporter gene, downstream from the inserted fragments. The recommended modification to the reverse transcription protocol were followed^3^: 2μg of poly A+ mRNA, 1 μL of 12.5μM primer per 20 μL reaction, and extension was performed for 60 min at 50°C. All reverse transcription reactions were cleaned up using a MinElute PCR Purification Kit (Qiagen #28004) and eluted in 15 μL of pre-heated dH2O. 8 PCR reactions were performed for each replicate sample to ensure output library complexity and to reduce potential PCR bias. PCR was performed using HPLC-purified primers (F: 5′-CAAGCAGAAGACGGCATACGAGAT-3′, R: 5′-AATGATACGGCGACCACCGAGATCTACAC[X8]TCGTCGGCAGCGTC-3′; “X8” sequence corresponds to sample barcode, chosen from Illumina Nextera barcode list) and KAPA HiFi HotStart PCR Kit (Kapa #KK2502). Thermocycler conditions were: 95°C for 3 min, then 5 cycles of: 98°C for 20 s, 63°C for 15s, and 72°C for 90 s. To reduce GC and size bias in PCR, the appropriate number of PCR cycles was determined using qPCR following the same thermocycler conditions for 35 cycles. PCR reactions were pooled and cleaned up with the MinElute PCR Purification Kit (Qiagen #28004) and submitted for next-generation sequencing (NGS) at the Hartwell Center for Bioinformatics and Biotechnology at St. Jude.

BasicSTARRseq (https://git.bioconductor.org/packages/BasicSTARRseq) was used to identify significant active sites using a max p-value cutoff of p<0.001 and a sample coverage cutoff of >=10. As a validation of these results, a DESeq2-based differential analysis approach was also implemented.^4^ In this approach, replicates of input and ATAC-STARR-seq RNA output were incorporated into the DESeq2 differential analysis comparing enrichment of RNA output (at 0hr, 6hr and 24hr) over input at all identified ATAC-seq sites in each cell line. Because true replicates of plasmid DNA input did not exist, ATAC-seq data for each replicate (1, 2 or 3) were pooled across the time course (e.g., replicate #1 for 0hr, 2hr, 6hr, 12hr and 24hr, etc.) to generate 3 pooled ATAC-seq replicates. Active STARR-seq sites from this contrast were identified across all ATAC-seq sites by a statistically significant enrichment (FDR< 0.05) of ATAC-STARR-seq RNA output signal over ATAC-seq input signal for each treatment condition (GC for 0hr, 6hr or 24hr).

**CRISPR/Cas9 genome editing**

*TLE1 and ROR1* gene knockdown pools were generated using CRISPR-Cas9 technology. Briefly, 1 million 697 or Nalm6 cells were transiently transfected with precomplexed ribonuclear proteins (RNPs) consisting of 300pmol of chemically modified sgRNA (Synthego; see Table 1 below for sgRNA sequences) and 100pmol of S*p*Cas9 protein (St. Jude Protein Production Core) via nucleofection (Lonza, 4D-Nucleofector™ X-unit) using solution P3 and program CA-137 (697) or CV-104 (Nalm6) in a large (100ul) cuvette according to the manufacturer’s recommended protocol. To validate disruptions, targeted amplicons were generated using gene specific primers with partial Illumina adapter overhangs and sequenced as previously described^5^. Cell pellets of approximately 10,000 cells were lysed and used to generate gene specific amplicons with partial Illumina adapters in PCR#1. Amplicons were indexed in PCR#2 and pooled with targeted amplicons from other loci to create sequence diversity. Additionally, 10% PhiX Sequencing Control V3 (Illumina) was added to the pooled amplicon library prior to running the sample on an Miseq Sequencer System (Illumina) to generate paired 2 X 250bp reads. Samples were demultiplexed using the index sequences, fastq files were generated, and NGS analysis was performed using CRIS.py^6^.

**Table 1**

| Target | sgRNA 1 | sgRNA 2 (HGR deletion only) |
| --- | --- | --- |
| *TLE1* | 5’- GGCACAGAUAAACGCAGAAA -3’ |  |
| *ROR1* | 5’ - GAGAUGUUCCAUGAUGAGGU -3’ |  |
| *TLE1* HGR variant | 5’- UUAGCAGAUUUUCCUUACCA -3’ | 5’- UCGCUGUAUGGCCCCUAAGG -3’ |
| *TLE1* HGR Peak1585 | 5’- UCGUGGUGCUUCACCUCGGA -3’ | 5’- UAUGAACUGCUUAGACUCUC -3’ |
| *ROR1* HGR Peak42 | 5’- ACAUGGAGAAAAGGAACCAC -3’ | AUAUAAAAAGCAGCCGCUGA -3’ |
| *ROR1* HGR Peak43 | 5’- UCACUGGUGUGUAAACUGUG -3’ | AAUAUAAGCUCAUGAUGUCA -3’ |

*HGR deletions* in 697 or Nalm6 were generated using CRISPR-Cas9 technology. In brief, one million 697 or Nalm6 cells were transiently transfected with precomplexed ribonuclear proteins (RNPs) consisting of 100pmol of each chemically modified sgRNA (Synthego), 35pmol of Cas9 protein (St. Jude Protein Production Core), and 3ug of ssODN (Alt-R modifications, IDT; see Table 2 below) via nucleofection (Lonza, 4D-Nucleofector™ X-unit) using solution P3 and program CA-137 (697) or CV-104 (Nalm6) in a large (100ul) cuvette according to the manufacturer’s recommended protocol. Three days post-nucleofection, genomic DNA was harvested via crude lysis and used for PCR amplification (see Table 2 below for primers). The presence of the desired deletion was confirmed via gel electrophoresis and sequencing.

**Table 2**

| **Target** | **ssODN** | **Primer F** | **Primer R** |
| --- | --- | --- | --- |
| *TLE1* HGR variant | tagagaggaatgcccaccttattttagcagattttccttaGAATTCaggagggatccaggactgactgtctgccctgcagtttgcc | ctgtctagagactgcctttccccc | acgcagggtggggatcaggaaagca |
| *TLE1* HGR Peak1585 | aaggaagacagggtaggagaggatcgtggtgcttcacctcGAATTCctcagggagaccctcagcttatccagctgtaaaaatcaaa | gcacagagtgtcagacgcggcacag | tgattcaaccaaccagtaggaggcaa |
| *ROR1* HGR Peak42 | agagcaggtttcgtctgctgttctttctcagacccctgtggaattctgatggtttttttacatttcaatttcattttgaggcttgg | ggagggaggcaaggctggagaacca | tcattcacacctcacagggtctccc |
| *ROR1* HGR Peak43 | attttaatggcatgcggtctttctcactggtgtgtaaactGAATTCcatcatgagcttatattctgttggggagtgacaaacaata | cctggctattagtgcccagagtgacc | cctccacctcatgtactggc |

**RT-qPCR**

Parental and HGR deleted 697 cells or HGR deleted Nalm6 cells were treated with prednisolone (697= 10uM, Nalm6= 5uM) or vehicle control for the specified time point (6hrs= *TLE1* HGR Peak1585; 24hrs= Nalm6 *TLE1* HGR variant, 697 *ROR1* HGR Peak42 and 697 *ROR1* HGR Peak43). Cells were then collected and resuspended in an RLT/BME mixture for total RNA isolation using RNeasy Midi Kit (Qiagen #75144). Subsequent cDNA synthesis was completed using the High-Capacity RNA-to-cDNA Kit (Applied Biosystems #4387406). RT-PCR reactions were prepared using the TaqMan Fast Advanced Master Mix (Applied Biosystems #4444557) and TaqMan Gene Expression Assays (TLE1: Hs00270768_m1, ROR1: Hs00938677_m1, TBP: Hs00427620_m1), and run on a QuantStudio 3 Real-Time PCR Instrument using the recommended TaqMan Fast Advanced Master Mix PCR conditions. Statistical significance was determined by a two-sided Student’s t-test and error bars denote standard deviation.

**CRISPR interference screening**

sgRNAs and control non-targeting sgRNAs (11038 targeting + 100 control) were designed and synthesized by Custom Array. Briefly, all the potential 20-bp sequences followed by the PAM sequences (NGG) at GR binding sites that mapped to GC-resistance accessible chromatin sites were identified. To maximize the dCas9 target specificity, we narrowed down the list by ensuring that one guide sequence and the PAM sequences are specifically mapped to one specific genomic loci. Finally, all the gRNAs with continued “TTTT” were removed from the library. The 100 non-targeting sgRNA sequences served as negative controls. Custom Array synthesized the sgRNA oligo library.

sgRNAs were amplified and purified (Qiagen PCR Purification Kit, #28104). gRNAs were cloned into the lentiviral pXPR_003-puro-IRES-CFP vector using Esp3i cut sites using Gibson Assembly Cloning Kit (NEB, #E5510S) and DH5a competent E. Coli cells (NEB, #C2987H). Cells were streaked across sixteen 20 cm x 25 cm agar plates with ampicillin and grown overnight at 37° C. The following day, the colonies were scrapped off the plate into an LB broth suspension and pooled and plasmids were purified (ThermoFisher: PureLink™ HiPure Plasmid Filter Maxiprep Kit, #K210016).

sgRNA plasmid libraries were subsequently packaged into viral particles. Hek293T cells were plated across twelve T175 flasks and let to grow to 80% confluence. Plasmid DNA (sgRNA plasmid library, CAG-kGP1-1R, CAG-RTR2, and HDM-G) was then transfected into the Hek293T cells using Lipofectamine LTX reagent (ThermoFisher, Cat #A12621) per manufacturer’s protocol in 15 mL serum free RPMI-1640 medium overnight. The following day, 15 mL of RPMI-1640 + 20% FBS medium was added and cells were incubated for 48 hours at 37° C and 5% CO_2_. Medium containing sgRNA plasmids packaged into viral particles was then collected. Ultracentrifugation was performed to concentrate the lentiviral library in 120mL media at 25000 RPMS for 2 hours at 4°C. Virus was resuspended in 1 mL of RPMI-1640. A 10uL aliquot of concentrated virus was used to verify the presence of viral particles using Lenti-X Go-Stix Plus (Takara Biosciences, #631281).

Spinfection was performed on 51 million single-cell derived clones of 697 or Nalm6 SID4X-dCas9-KRAB expressing cells at a low multiplicity-of-infection (MOI) to maintain >1:1000 sgRNA:cell ratio for equal representation of sgRNAs and to ensure each cell received only 1 sgRNA plasmid. Cells were pooled together, centrifuged at 500xg, washed with PBS, resuspended in complete RPMI-1640 and transferred to a T75 flask. After 24 hours, cell were placed under puromycin selection and neomycin selection in media to ensure sgRNA and SID4X-dCas9-KRAB construct expression media. Cells remained under selection for 2 weeks until reaching >90% viability. sgRNA expression was further confirmed by CFP expression.

After expansion of cells, 40 million 697 or Nalm6 SID4X-dCAs9-KRAB + sgRNA expressing cells were plated into a T75 in complete medium in triplicate across five treatment conditions. Control cells were given equal volume of saline (Vehicle Control) and treatment groups were given 10uM prednisolone (697) or 5uM prednisolone (Nalm6). Cells incubated with drug for 72 hours and subsequently cultured for 5-7 doubling times to allow surviving population to expand. After recovery, 50 million cells were collected and spun at 500xg for 5 minutes, washed once with PBS, and genomic DNA was isolated (Qiagen, Blood and Cell Culture DNA Maxi Kit, Cat #13362).

sgRNA amplification from gDNA was performed as previously described^7^. Briefly, gDNA samples were divided among multiple 100uL PCR reactions with each reaction having up to 10ug of gDNA. PCR amplification was carried out using Titanium Taq (Takara) with 5% DMSO.

The sgRNA sequencing data were de-barcoded and trimmed to contain only the sgRNA sequence and subsequently mapped to the reference sgRNA library without allowing for any mismatches and sequence counts were obtained for all sgRNAs. For each cell line, only sgRNAs mapping to HGRs were used for sgRNA enrichment and ranking analysis. DESeq2^4^ was used to identify significant enrichments of individual sgRNAs and MAGeCK^8^ was used to subsequently aggregate sgRNAs at each site of GR occupancy to determine overall enrichment at each site. All HGRs that harbored positive log_2_ enrichment from sgRNA aggregation (i.e. greater enrichment in prednisolone treated cells versus vehicle control treated cells) were used in downstream integrative analyses.

**Drug viability assays**

Drug viability assays were performed as previously outlined^9, 10^. Cells were seeded at 20,000 cells per well in a 96-well plate and treated with prednisolone at various concentrations as previously described^10^. Following a 72-hour incubation, ALL cell line viability was measured using the Cell Titer-Glo Luminescent Cell Viability Assay (Promega, G7571). Concentration of prednisolone used are provided in figure legends for GC drug response curves. Cells were also treated with vehicle to control for potential confounding effects on cell viability and growth from gene disruptions or regulatory element deletions. For primary ALL cells, following a 96-hour incubation, MTT (3-4, 5-dimethylthiazol-2,5-diphenyl tetrazolium bromide) was added to wells to measure viability. The drug concentration that killed 50% of the leukemia cells (LC50 value) was determined with a dose-response model. GC-resistant primary ALL cells were defined as having an LC50 of 64μM or greater, whereas GC-sensitive primary ALL cells were defined as having an LC50 of less than 0.1μM. For HGR knockout studies, a single concentration of prednisolone was used and is provided in figure legends. Statistical significance was determined by a two-sided Student’s t-test and error bars denote standard error of the mean.

**Data analysis**

To identify sites of GR occupancy that harbor GREs and related motifs, we combined two strategies. We identified sites of GR occupancy in our ChIP-seq data that harbored GREs using MEME-ChIP^11^. Additionally, we used HOMER motif software^12^ to identify all genome-wide locations of GREs (HOMER motifs 146 and 117), half-site motifs (Motifs: M11124, M00192, M00921 and M00960) and nGRE sequences (CTCCN_(0-2)_GGAGA) previously identified^13^ using recommended motif stringency cutoffs. We subsequently intersected the locations of all GR motifs with our GR ChIP-seq data to identify sites of GR occupancy that contained discernable GR motifs. Super-enhancers were identified using Rank Ordering of Super-Enhancers (ROSE)^14, 15^. HiChIP loops were identified using FitHiChIP^1^. TOBIAS was used to identify TF footprints^16^. Paired Expression and Chromatin Accessibility data (PECA)^17^ was used to generate TF-target connections. Active ATAC-STARR-seq sites were identified using BasicSTARRseq (<https://git.bioconductor.org/packages/BasicSTARRseq>) or DESeq2^4^. Variants associated with GC resistance in primary ALL cells were previously identified by genome-wide association studies were previously performed by our group using SNP genotyping data and *ex vivo* LC50 GC resistance results obtained from primary ALL cell samples from patients on St. Jude Total Therapy XVI^9^. We associated SNPs to GC LC50 values (log-transformed) in primary ALL cells using linear regression assuming a genetic additive effect. GC-resistance associated SNPs with a minor allele frequency (MAF) >= 1% in at least one 1000 Genomes population were considered, along with all SNPs in high LD (r^2^>=0.8). RNA-seq results from normal B-cells were obtained from publicly available data^18^

**REFERENCES**

1. Bhattacharyya S, Chandra V, Vijayanand P, Ay F. Identification of significant chromatin contacts from HiChIP data by FitHiChIP. *Nat Commun* 2019 Sep 17; **10**(1)**:** 4221.

2. Muerdter F, Boryn LM, Woodfin AR, Neumayr C, Rath M, Zabidi MA*, et al.* Resolving systematic errors in widely used enhancer activity assays in human cells. *Nat Methods* 2018 Feb; **15**(2)**:** 141-149.

3. Wang X, He L, Goggin SM, Saadat A, Wang L, Sinnott-Armstrong N*, et al.* High-resolution genome-wide functional dissection of transcriptional regulatory regions and nucleotides in human. *Nat Commun* 2018 Dec 19; **9**(1)**:** 5380.

4. Love MI, Huber W, Anders S. Moderated estimation of fold change and dispersion for RNA-seq data with DESeq2. *Genome Biol* 2014; **15**(12)**:** 550.

5. Sentmanat MF, Peters ST, Florian CP, Connelly JP, Pruett-Miller SM. A Survey of Validation Strategies for CRISPR-Cas9 Editing. *Sci Rep* 2018 Jan 17; **8**(1)**:** 888.

6. Connelly JP, Pruett-Miller SM. CRIS.py: A Versatile and High-throughput Analysis Program for CRISPR-based Genome Editing. *Sci Rep* 2019 Mar 12; **9**(1)**:** 4194.

7. Hanna RE, Hegde M, Fagre CR, DeWeirdt PC, Sangree AK, Szegletes Z*, et al.* Massively parallel assessment of human variants with base editor screens. *Cell* 2021 Feb 18; **184**(4)**:** 1064-1080 e1020.

8. Li W, Xu H, Xiao T, Cong L, Love MI, Zhang F*, et al.* MAGeCK enables robust identification of essential genes from genome-scale CRISPR/Cas9 knockout screens. *Genome Biol* 2014; **15**(12)**:** 554.

9. Autry RJ, Paugh SW, Carter R, Shi L, Liu J, Ferguson DC*, et al.* Integrative genomic analyses reveal mechanisms of glucocorticoid resistance in acute lymphoblastic leukemia. *Nat Cancer* 2020 Mar; **1**(3)**:** 329-344.

10. Paugh SW, Bonten EJ, Savic D, Ramsey LB, Thierfelder WE, Gurung P*, et al.* NALP3 inflammasome upregulation and CASP1 cleavage of the glucocorticoid receptor cause glucocorticoid resistance in leukemia cells. *Nat Genet* 2015 Jun; **47**(6)**:** 607-614.

11. Bailey TL, Boden M, Buske FA, Frith M, Grant CE, Clementi L*, et al.* MEME SUITE: tools for motif discovery and searching. *Nucleic Acids Res* 2009 Jul; **37**(Web Server issue)**:** W202-208.

12. Heinz S, Benner C, Spann N, Bertolino E, Lin YC, Laslo P*, et al.* Simple combinations of lineage-determining transcription factors prime cis-regulatory elements required for macrophage and B cell identities. *Mol Cell* 2010 May 28; **38**(4)**:** 576-589.

13. Surjit M, Ganti KP, Mukherji A, Ye T, Hua G, Metzger D*, et al.* Widespread negative response elements mediate direct repression by agonist-liganded glucocorticoid receptor. *Cell* 2011 Apr 15; **145**(2)**:** 224-241.

14. Whyte WA, Orlando DA, Hnisz D, Abraham BJ, Lin CY, Kagey MH*, et al.* Master transcription factors and mediator establish super-enhancers at key cell identity genes. *Cell* 2013 Apr 11; **153**(2)**:** 307-319.

15. Loven J, Hoke HA, Lin CY, Lau A, Orlando DA, Vakoc CR*, et al.* Selective inhibition of tumor oncogenes by disruption of super-enhancers. *Cell* 2013 Apr 11; **153**(2)**:** 320-334.

16. Bentsen M, Goymann P, Schultheis H, Klee K, Petrova A, Wiegandt R*, et al.* ATAC-seq footprinting unravels kinetics of transcription factor binding during zygotic genome activation. *Nat Commun* 2020 Aug 26; **11**(1)**:** 4267.

17. Duren Z, Chen X, Jiang R, Wang Y, Wong WH. Modeling gene regulation from paired expression and chromatin accessibility data. *Proc Natl Acad Sci U S A* 2017 Jun 20; **114**(25)**:** E4914-E4923.

18. Calderon D, Nguyen MLT, Mezger A, Kathiria A, Muller F, Nguyen V*, et al.* Landscape of stimulation-responsive chromatin across diverse human immune cells. *Nat Genet* 2019 Oct; **51**(10)**:** 1494-1505.
